# Dynamic Activation of NADPH Oxidases in Immune Responses Modulates Differentiation, Function, and Lifespan of Plasma Cells

**DOI:** 10.1002/eji.202350975

**Published:** 2025-02-11

**Authors:** Olivia T. M. Bucheli, Daniela Rodrigues, Carolin Ulbricht, Anja E. Hauser, Klaus Eyer

**Affiliations:** ^1^ ETH Laboratory for Functional Immune Repertoire Analysis Institute of Pharmaceutical Sciences, D‐CHAB, ETH Zürich Zürich Switzerland; ^2^ Department of Rheumatology and Clinical Immunology Charité ‐ Universitätsmedizin Berlin corporate member of Freie Universität Berlin and Humboldt‐Universität zu Berlin Berlin Germany; ^3^ Immune Dynamics Deutsches Rheuma‐Forschungszentrum (DRFZ) a Leibniz Institute, Charitéplatz 1 Berlin Germany; ^4^ Department of Biomedicine Aarhus University Aarhus C Denmark

**Keywords:** B cells, BCR signaling, immune metabolism, immune response, NADPH oxidase, TLR4 signaling

## Abstract

NADPH‐oxidase (NOX)‐derived reactive oxygen species (ROS) have been described to play essential roles in B‐cell activation processes. However, several key questions concerning NOX activity and subsequent ROS production remain unaddressed, including fundamental processes such as differentiation, functional competence, cellular metabolism, and viability. This study investigated these questions in a murine B‐cell response after secondary immunization. We combined single‐cell transcriptomics and single‐cell detection of NOX activity and observed that various subsets of B cells dynamically express *NOX1* and *NOX2*. The NOX^+^ cellular phenotype correlated with increased activity of metabolic pathways, augmented lactate production, lower IgG secretion rates, and markers for longevity. The NOX^+^ cellular phenotype was also associated with increased cellular stress and apoptosis, underscoring the intricate relationship between ROS and cellular survival. Consequently, these insights advance our understanding of how long‐lived humoral immunity is formed.

## Graphical abstract

NADPH‐oxidase (NOX)‐derived reactive oxygen species (ROS) play key roles in B‐cell activation. This study, using murine models, revealed dynamic NOX1/2 expression linked to metabolic activity, lactate production, reduced IgG secretion, stress, and apoptosis, highlighting ROS's impact on B‐cell survival and long‐lived humoral immunity.

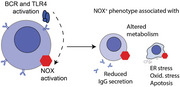

## 1 Introduction

Antigen challenge induces a highly dynamic process that leads to a humoral immune response by developing antigen‐specific plasma cells (PCs), which secrete antibodies. A swift and thorough activation of naïve B cells or preformed antigen‐specific memory B cells (MBCs) is crucial for this process and was reported to occur in secondary lymphoid organs such as the spleen. Upon activation, the induced signaling events lead to an initial clonal expansion of B cells, followed by their differentiation into extrafollicular PCs, new MBCs, or germinal center B cells (GCBCs) [1, 2, 3, 4]. With help from T cells, signaling from the B‐cell receptor (BCR) and co‐stimulatory receptors such as Toll‐like receptors (TLRs) were shown to induce the activation of B cells, and a careful balance of the signaling strength was described to be a differentiator between activation [5, 6, 7] and inhibition [8, 9, 10] of cell functionality. Indeed, several studies found that excessive BCR signaling decreased B‐cell survival and was associated with autoimmune diseases and malignancies [9, 11, 12], while insufficient signaling did not induce B‐cell activation [13]. Thus, maintaining a BCR signaling balance remains important for appropriate B‐cell activation, survival, control, and differentiation. On their path to becoming long‐lived antigen‐specific PCs, the formed GCBCs undergo a specific program of affinity maturation and selection, which occur in two distinct microanatomical zones: the dark zone (DZ), where cells undergo cell division and somatic hypermutation [14, 15], and the light zone (LZ), where positive selection occurs. The outcomes of the process in the LZ are the cell re‐entry into the DZ for further rounds of mutation, apoptosis, or differentiation into MBCs, plasmablasts, or immature PCs that further differentiate into mature PCs or become apoptotic and are removed [16, 17, 18, 19]. Due to these processes, affinity‐matured PCs are expected to display increased affinity toward their antigen. The generation of reactive oxygen species (ROS) has been reported as crucial second messengers for signal transduction through various receptors, including BCR and TLR4 [20]. ROS involvement in B‐cell signaling was first demonstrated by hydrogen peroxide treatment, inducing BCR‐like signaling patterns [21]. Moreover, ROS have been reported to regulate protein tyrosine phosphatases [22, 23], kinases [22], transcription factors [23], and calcium channels [24], all involved in cell signaling events. Subsequent studies in other cell types further showed that ROS regulated signaling by reversibly oxidizing various molecules, such as inhibitory protein tyrosine phosphatases [25, 26], thereby augmenting signaling. Whether similar mechanisms might also be present in B cells has not been resolved. NADPH oxidases (NOXes) and the mitochondrial respiratory chain were identified as the two major sources of ROS production in B cells [20]. In mouse splenic B cells, four of the six known NOX isoforms were observed, namely NOX1‐3 and DUOX2 [24, 27], while NOX4 and DUOX1 have not been reported to be expressed.

For the enzymatic activity, all NOX isoforms were shown to require the formation of a complex consisting of their transmembrane protein subunit with cytosolic proteins [28, 29]. Active NOXes were found to produce superoxide anions (O_2_^•−^) from NADPH and oxygen [28, 29], whereby the superoxide anions were further converted to hydrogen peroxide [30]. Two phases of ROS production upon BCR ligation were identified: an early phase within one hour mediated by NOX2, followed by a stronger and more sustained production of ROS after two to six hours, whereby their source remains a question for debate [24, 27, 31]. Apart from modulating BCR signaling, various other functions of ROS have been identified in the activation, proliferation, and survival of BCR‐stimulated B cells [21, 24, 27, 31, 32, 33].

BCR and TLR4 signaling were shown to not only induce ROS production but also to alter cellular metabolism [34, 35, 36, 37, 38]. Reports further suggested that changes in cellular metabolism were not only a direct consequence of cell state but also a driving factor of differentiation, function of B cells, as well as the longevity of plasma cells (reviewed in [39]). Furthermore, links between ROS and cellular metabolism have already been demonstrated, including the ROS‐induced inactivation of the tricarboxylic acid (TCA) cycle enzyme aconitase which subsequently forces the cells to switch from oxidative phosphorylation (OXPHOS) to lactate metabolism [40]. The reports showed, in summary, that ROS are required for balanced and prolonged BCR signaling as well as cell function and survival. Moreover, the implications of unbalanced ROS in differentiation into PCs and antibody production have been shown in the literature [24, 41, 42, 43, 44, 45], including a link between dysregulation of B‐cell activation, NOX activity, and autoimmune diseases.

While the involvement of NOX‐generated ROS in the process of B‐cell activation has been established, there remain unresolved questions regarding the fundamental mechanisms, kinetics, and implications of NOX activity. Likewise, ROS generation plays a role in differentiation, functional capacity, cellular metabolism, and viability, as B‐cell activation represents merely the initial phase within the complex trajectory of cellular differentiation toward long‐lived, functional PCs. Consequently, this study undertook a comprehensive investigation encompassing transcriptomics and a concurrent direct evaluation of active NOXes at the single‐B‐cell level across various subsets within the B‐cell lineage found in the spleen postexposure to antigens. Our primary focus evolved around delineating B‐cell activation and differentiation kinetics as they transition into PCs, specifically considering NOX and ROS dynamics within a murine model and the consequences for metabolic processes, cellular functionalities, and viability.

## 2 Results

### 2.1 Activated, Germinal Center, Memory B, and Plasma Cells Express NOX1 and NOX2, and Expression Varies Dynamically Upon Activation

**FIGURE 1:**
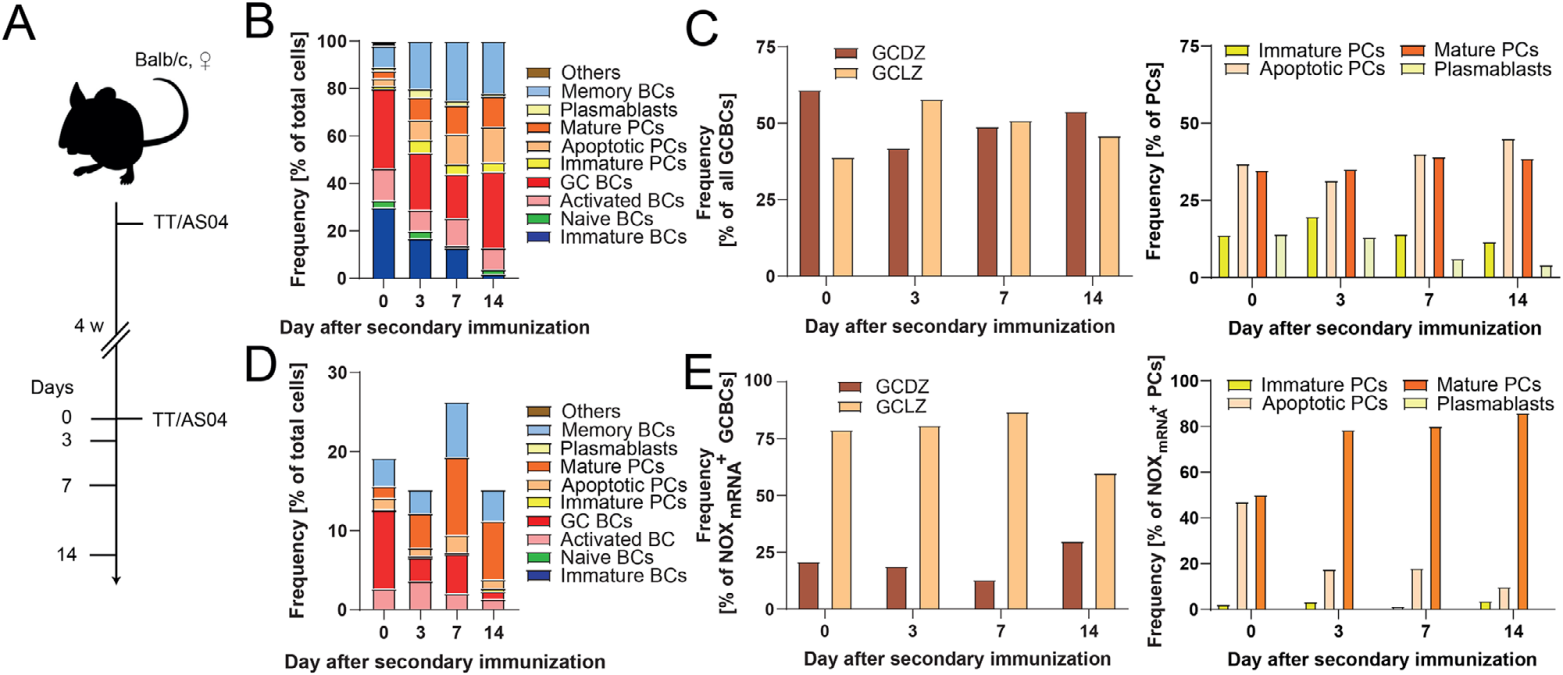
B cell populations and NOX expression measured by scRNA‐Seq. (A) Employed immunization scheme. (B) Frequency of B‐cell subpopulations; immature B cells (blue), activated B cells (pink), naïve B cells (green), GCBCs (red), PCs divided into immature (dark yellow), apoptotic (light orange), mature PCs (dark orange) and plasmablasts (yellow), and MBCs (blue) throughout the immune response to TT supplemented with MPLA in alum (called AS04). Non‐B cells are summarized under others (brown). (C) Frequency of GCDZ and GCLZ cells of all GCBCs (left) and of immature, apoptotic, and mature PCs and plasmablasts (right). (D) The total frequency of NOX_mRNA_^+^ B cells in relation to all B cells throughout the immune response and the division into subpopulations. Immature B cells, naïve B cells and immature PCs were NOX_mRNA_^−^. (E) Frequency of NOX_mRNA_^+^ GCBCs in GCDZ and LZ (left), and of immature, apoptotic, and mature PCs and plasmablasts related to all NOX_mRNA_^+^ mature PCs (right). All plasmablasts were NOX_mRNA_^−^. For this analysis, splenic B cells from two mice were mixed in a 1:1 ratio followed by scRNA‐Seq (*n* = 800–5500 cells).

First, we assessed the expression of NOXes in splenic B cells during an immune response by single‐cell transcriptomic analysis. Female BALB/c mice were immunized twice with tetanus toxoid (TT) in alum with monophosphoryl lipid A (MPLA), called adjuvant system 04 (AS04), with an interval of four weeks (Figure 1A). Splenic B cells were isolated after secondary immunization on days 0 (i.e., on the day of secondary immunization but without immunization, see methods for more detail), 3, 7, and 14. The purified B cells from two mice were pooled in a 1:1 ratio, based on total cell numbers, for mRNA sequencing, and the splenic B cells were classified into immature B cells, naïve B cells, activated B cells, GCBCs, MBCs, and immature and mature PCs. First, 98–100% of sequenced cells belonged to the B‐cell lineage and were classified into one of these categories using the markers from Table S1 (Figure S1 shows clusters). Regarding B‐cell subpopulations, the relative composition of the analyzed cell samples throughout the immune response was highly dynamic (Figure 1B). The frequency of immature B cells decreased steadily after the re‐immunization, reaching 0% on day 14, while the activated B cells remained relatively constant during the immune response (11 ± 2%). Naïve cells were also present throughout the analysis, forming between 1% and 3% of the analyzed cells. The relative frequency of GCBCs decreased shortly after the booster immunization (34% on day 0, 24% on day 3, and 19% on day 7) before rebounding again on day 14 (32%), whereas the frequency of both PCs and MBCs was dynamic throughout the immune response, starting with 9% (both) and reaching 33% and 22% on day 14, respectively. Additionally, we identified the main cell subsets within the GCBC population, namely dark zone and light zone GC cells (GCDZ and GCLZ cells, respectively), as well as those of PCs, namely immature, apoptotic, and mature PCs and plasmablasts based on key gene markers characteristic of these subpopulations (Table S1) [46, 47, 48, 49, 50]. Comparing the relative frequencies of GCDZ and GCLZ cells, we found a 60/40 split on day 0, with the majority belonging to GCDZ cells (61%) (Figure 1C). The ratios between GCDZ and GCLZ cells differed over the days, with GCLZ cells being the bigger subset on day 3 (60/40 ratio) and close to 1:1 on days 7 and 14. Regarding PCs, mature PCs and apoptotic PCs represented the majority of all PCs (75 ± 8% over all days, whereby the ratio was approx. 1:1) on all days, followed by immature PCs (15 ± 4%) and plasmablasts (9.9 ± 6.1%, Figure 1C).

Next, the expression changes of differentially expressed genes (DEGs) for all B‐cell subpopulations were investigated, including those of NOXes (up‐ or downregulated). We specifically analyzed expression changes of genes encoding for NOX1‐4 and DUOX1‐2 in the B‐cell subpopulations over time (Figure 1D; Figure S2). A dynamic change was observed, whereby antigen re‐exposure first led to a decrease in B cells with upregulated gene expression (all NOX isoforms), termed NOX_mRNA_^+^ B cells, and then, on day 7, to a peak (26%) that subsequently digressed, hence displaying an initial collapse followed by expansion and collapse. NOX_mRNA_^+^ cells were part of all subpopulations except immature B cells, naïve B cells, and immature PCs, and the frequency varied over subpopulations and time. The fraction of NOX_mRNA_^+^ cells within the activated B cells was low on the day of re‐immunization, peaked after the booster on day 3 (40%), and decreased to around its initial value on later days (16.5% and 13% on days 7 and 14, respectively). The fractions of NOX_mRNA_^+^ cells within GCBCs, MBCs, and mature PCs were similarly evolving, collapsing, and expanding, along the immune response, with distinct maxima on days 0 and 7. Furthermore, we assessed whether NOX_mRNA_^+^ GCBCs were either GCDZ or GCLZ cells, and NOX_mRNA_^+^ PCs subsets as defined in Table S1 (Figure 1E). Our results showed that NOX_mRNA_^+^ GCBCs mostly corresponded to GCLZ cells at all investigated time points (60–87%). Additionally, except for day 0, we observed that most NOX_mRNA_^+^ PCs displayed gene expression signatures linked to mature PCs (≥78%), especially on the later days of analysis. No NOX_mRNA_^+^ plasmablasts were observed, and only a small fraction of NOX_mRNA_^+^ immature and apoptotic PCs were observed after day 0. To confirm this observation and gain further insights into the individual subsets, we reversed the analysis (Figure S3). Between half and up to 81% of mature PCs were found NOX_mRNA_^+^, whereas the NOX_mRNA_^+^ PCs were always in the minority in apoptotic and immature PCs (Figure S3).

Moreover, the same analysis was performed in PCs extracted from the bone marrow (BM). We found only mature and apoptotic PCs in the BM. As expected, we found a higher percentage of cells displaying a mature PC signature in BM on all days (≥92%, Figure S4A). As observed in the spleen, the NOX_mRNA_^+^ PCs were enriched within the mature PC subpopulation (≥95%, Figure S4B). On the contrary, the relative frequency of NOX_mRNA_^−^ mature PCs increased over time, indicating a shift away in NOX expression in the BM (Figure S4C). In the small percentage of apoptotic PCs, most cells were NOX_mRNA_^−^ (Figure S4D), also in line with the splenic apoptotic PCs.

Lastly, we investigated which NOX isoforms were expressed by the NOX_mRNA_^+^ B cells (Figure S5). All respective B‐cell subpopulations expressed NOX1 and NOX2, whereas no expression of NOX3, NOX4, DUOX1, and DUOX2 was detected in any analyzed B cell. The relative frequency of cells expressing NOX2 was higher than NOX1 on all days; on average, 1.3× more cells expressed NOX2 than NOX1. We further observed that the cells expressed either NOX1 or NOX2, with only a small percentage being double positive. The NOX isoform expression was not specific to subpopulations as all B‐cell subpopulations containing NOX_mRNA_^+^ cells showed expression of both isoforms (Figure S5).

### 2.2 NOX Activity Detection Reveals Distinct and Different Dynamics Compared with mRNA Analysis

**FIGURE 2:**
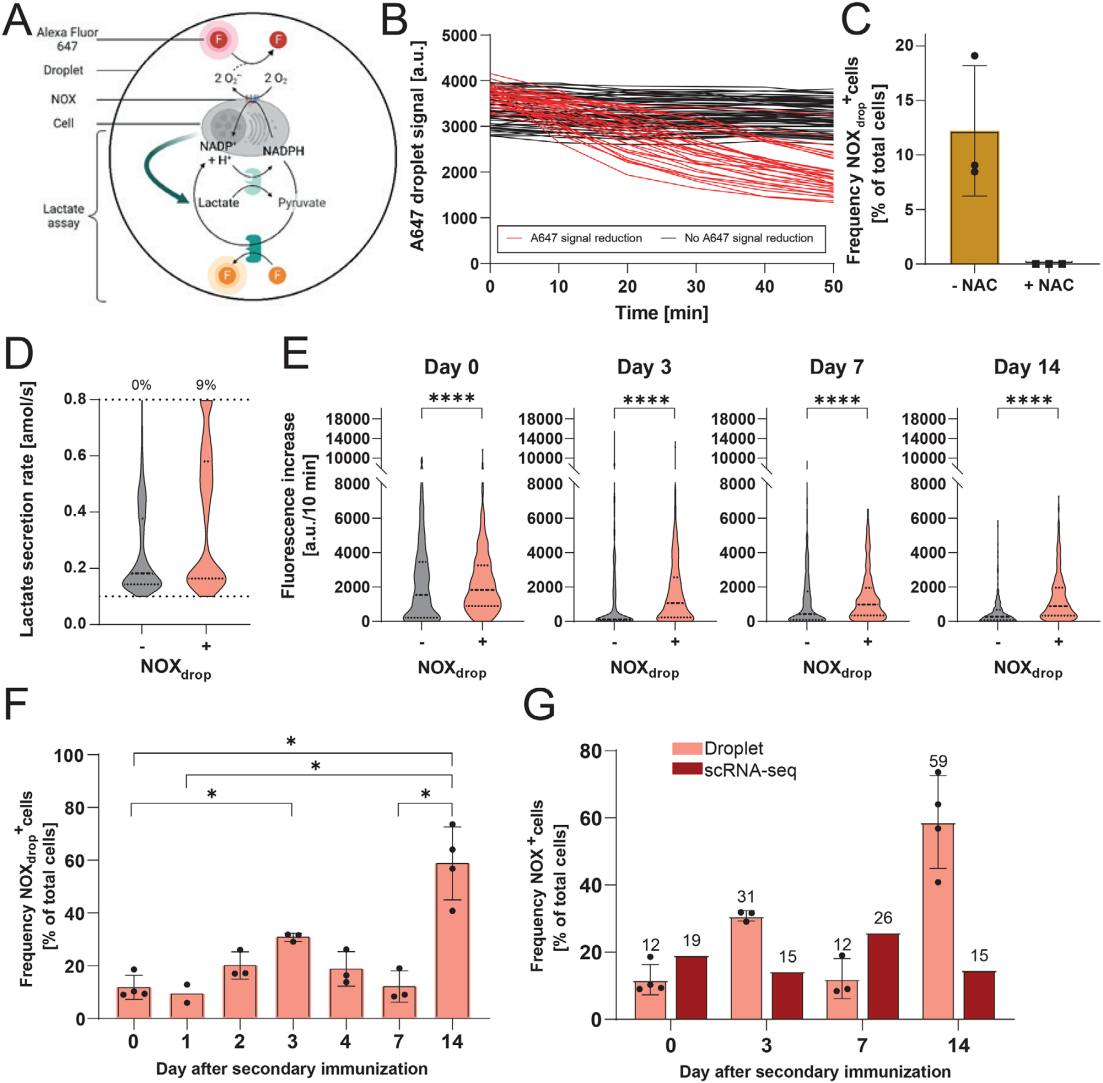
(A) Schematic representation of the bioassay to assess the presence of active NOXes. In short, NOXes catalyzed the conversion of molecular oxygen (O_2_) and NADPH into the ROS superoxide anion (O_2_^•−^), which decreased the fluorescence of extracellular A647. (B) Traces of A647 signal of droplets containing a B cell. Only in the presence of a cell with active NOXes, a reduction of the signal of the oxidation‐sensitive A647 was observed over the measurement time (highlighted in red, termed NOX_drop_^+^). (C) Frequency of NOX_drop_^+^ cells without or in the presence of the ROS scavenger NAC on day 7 (*n* = 3). (D) Distribution of lactate secretion rates from NOX_drop_^−^ and NOX_drop_^+^ cells (*n* = 1500, randomly selected in groups of 125 from each mouse and day of analysis). The dotted lines indicate the quantitative range of the lactate assay (0.1–0.8 amol/s [38]), and the number shown above the cutoff represents the percentage of B cells with a lactate secretion rate of ≥0.8 amol/s. (E) Comparison of the intracellular ROS level distributions between NOX_drop_^−^ and NOX_drop_^+^ cells. The fluorescence increase, related to ROS production, was measured using CellROX Green [38] (all *n* = 375, randomly selected in groups of 125 from each mouse). (F) Frequency of cells detected as NOX_drop_^+^ throughout the immune response (*n* = 2–4). (G) Comparison of frequency of NOX_drop_^+^ cells determined by droplet measurements (light red) and NOX_mRNA_^+^ cells detected by transcriptomic analysis (dark red, data from Figure 1C) throughout the immune response. The median, 25 and 75 quartiles are indicated as dashed and dotted lines, respectively, in the violin plots, and the mean and SD are displayed in the bar charts. The level of statistical significance is denoted as *p < 0.05, **p < 0.01, ***p < 0.001, and ****p < 0.0001. The schematic representation of the bioassay was created with BioRender.com.

As we observed a dynamic expression of NOXes in splenic B cells along the immune response, we aimed to study whether these enzymes were in their active state. Therefore, an assay to measure ROS production by NOXes was developed and introduced into microfluidic droplets. ROS produced by NOXes were detected by the fluorescence decrease of the oxidation‐sensitive fluorophore Alexa Fluor 647 (A647, Figure 2A) in the presence of an enzymatic assay replenishing NAPDH, which is used by NOXes to produce the ROS superoxide anion (O_2_^•−^). The resulting ROS oxidized A647, leading to a detectable loss of its characteristic fluorescence signal (Figure 2B). In the absence of the enzymatic assay, no reduction of the droplet's A647 signal was observed during the studied period (data not shown), indicating a negligible contribution of mitochondrial ROS. No decrease in fluorescence was found in droplets containing no cells (data not shown) demonstrating the containment of the signal loss within the single droplet and, thus, single‐cell resolution. Furthermore, the addition of the ROS scavenger *N*‐acetylcysteine (NAC) inhibited the loss of fluorescence, reducing the frequency of droplets displaying a signal loss from 12.1 ± 6.0% to 0.0 ± 0.0% in murine splenic samples (*n* = 3, Figure 2C). Since the enzymatic assay for detecting active NOXes depended on the lactate secretion of the cells to replenish NADPH, we investigated whether a specific lactate secretion rate was required for detecting active NOXes. For this purpose, a uniform random selection of cells from days 0 to 14 was pooled and divided into NOX_drop_^−^ and NOX_drop_^+^ based on the absence or presence of signal loss. The extracted lactate secretion rates were compared between the two groups (Figure 2D). In short, NOX activity, that is, A647 signal loss, was independent of lactate secretion rate as we observed lactate secretion rates throughout the quantitative range (0.1–0.8 amol/s [38]) in both subpopulations. The presence of low lactate‐secreting cells in the NOX_drop_^+^ population suggested that these rates were sufficient to replenish NADPH for the active NOX detection assay to function. Lastly, we aimed to compare our assay with a commercial assay to measure intracellular ROS independent of its source. To do so, we additionally added, as described in Bucheli et al. [38], the intracellular ROS bioassay comprising a cell‐permeable, ROS‐sensitive reagent (see methods) and compared the distribution of the ROS levels between NOX_drop_^−^ and NOX_drop_^+^ cells during the immune response (Figure 2E). Indeed, NOX_drop_^+^ cells displayed higher intracellular fluorescence and, thus, ROS production levels throughout the immune response, with a 1.2 to 8.8 times higher median value than their counterpart (*p*‐value <0.0001 for all days). Summarized, these controls indicated that the assay could identify cells with activated NOXes.

After the assay introduction and characterization, we employed the novel A647 signal loss method to detect active NOXes to monitor the frequency of NOX_drop_^+^ B cells throughout the secondary immune response to TT/AS04 (*n* = 2–4, Figure 2F). We added additional data generated for postimmunization days 1, 2, and 4 to resolve the NOX activation dynamics. On the day of re‐immunization (day 0), 12 ± 5% of extracted splenic B cells displayed active NOXes. The frequency followed a dynamic pattern with an intermediary peak observed on day 3 (31 ± 2%, *p*‐value 0.014 for comparison of day 3 to day 0), followed by a decrease on day 7 (12 ± 6%) and an absolute peak on day 14 (59 ± 14%, *p*‐values 0.028, 0.029, and 0.035 for comparison of day 14 to days 0, 1, and 7, respectively). Regarding active NOXes, we observed a dynamic in frequency consisting of expansion, collapse, and expansion. When directly compared with the transcriptomic data (Figure 2G), the frequencies of NOX_drop_^+^ B cells trended in the opposite direction and did not correlate (*R* ^2^, 0.51).

### 2.3 BCR and TLR4 Signaling Correlate with NOX Expression and Phenotype

Based on the dynamics of NOX_mRNA_^+^ and NOX_drop_^+^ B cells and the described activation of NOXes by BCR and TLR4 signaling pathways [20], we next assessed the contribution of BCR and TLR4 signaling toward NOX activation. For this purpose, we analyzed the BCR and TLR4 signaling pathways of NOX_mRNA_^−^ and NOX_mRNA_^+^ cells. For each pathway, only DEGs (*p*‐value <0.05) obtained after comparison of NOX_mRNA_^−^ and NOX_mRNA_^+^ cells to the pooled B cells from all time points were considered, indicating a positive or negative modulation of the pathways, depending on upregulation or downregulation of DEGs, respectively. Across all B‐cell differentiation stages, BCR and TLR4 signaling pathways were positively modulated in NOX_mRNA_^+^ B cells (Figures S6–S10), whereas they were either negatively or positively modulated to a lower extent in NOX_mRNA_^−^ B cells, with a few exceptions only.

**FIGURE 3:**
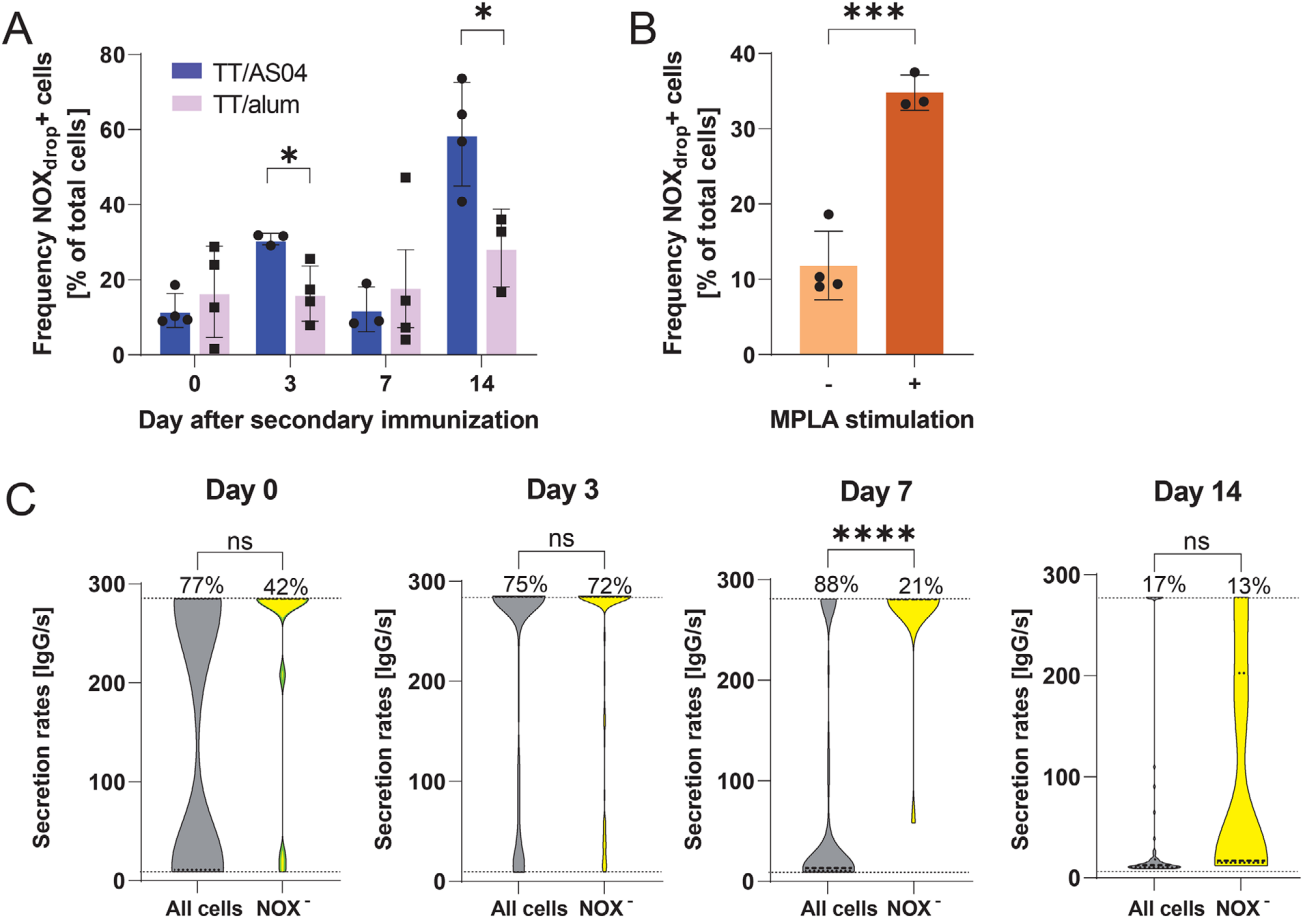
(A) Frequency of NOX_drop_^+^ cells obtained by immunization with TT/AS04 (purple) and TT/alum (pink) throughout the immune response. Mean, SD, and individual measurements (*n* = 3–4 mice) are shown. TT/AS04 data is identical to the data displayed in Figure 2G. (B) Frequency of NOX_drop_^+^ cells on day 0 without (−, orange) or with (+, red) ex vivo stimulation using MPLA (*n* = 3–4). (C) Distribution of measured IgG secretion rates of all cells (i.e., NOX independent; grey) or NOX_drop_^−^ cells (yellow) on days 0, 3, 7, and 14. The IgG secretion rate of NOX_drop_^+^ cells could not be determined due to the loss of the A647 signal. All IgG‐SCs from three mice per day were pooled. Day 0: *n*_all_ IgG‐SCs (grey) = 12, *n*_NOX_^−^ IgG‐SCs (yellow) = 13, day 3: *n*_all_ IgG‐SCs = 459, *n*_NOX_^−^ IgG‐SCs = 106, day 7: *n*_all_ IgG‐SCs = 78, *n*_NOX_^−^ IgG‐SCs = 17, day 14: *n*_all_ IgG‐SCs = 75, *n*_NOX_^−^ IgG‐SCs = 6. The dotted lines represent the quantitative range (9–285 IgG/s) and the number above the upper limit, the frequency of cells with an IgG secretion rate of ≥285 IgG/s of the respective category. The level of statistical significance is denoted as *p < 0.05, ***p < 0.001, and ****p < 0.0001.

After identifying a potential link between NOX upregulation and BCR/TLR4 signaling transcriptionally, we aimed to study their influence on the functional side by modifying the immunization. First, the influence of the adjuvant MPLA, the employed TLR4 agonist, was investigated by comparing mice immunized with TT/AS04 and TT/alum, that is, omitting MPLA, during the immune response (Figure 3A). While the TT/AS04 immunized mice displayed an intermediary peak of the frequency of cells displaying active NOXes on day 3 and an absolute peak on day 14, TT/alum immunized mice displayed a more stable frequency of NOX_drop_^+^ cells over the observed period, only showing a small increase on day 14. Indeed, a significant difference in the frequency of NOX_drop_^+^ cells obtained by immunizations with TT/AS04 and TT/alum was observed on days 3 and 14 (Figure 3A, *p*‐value 0.03 and 0.02, respectively), where TT/AS04 displayed around a two‐fold higher frequency of NOX_drop_^+^ cells. As these maxima seemed to be due to the addition of MPLA, as they were not observed using TT/alum, we wanted to study further the relationship between MPLA and NOX activity. Hence, cells from day 0 were stimulated *ex vivo* with MPLA. The NOX_drop_^+^ cell frequency increased significantly (Figure 3B, *p*‐value 0.0005), supporting the observed relationship between NOX activity and TLR4 signaling. To study the influence of BCR signaling, the frequencies of NOX_drop_^+^ cells from spleen samples of B18^hi^YellowCaB mice 5–7 days after a third immunization with 4‐hydroxy‐3‐nitrophenylacetyl‐chicken gamma globulin (NP‐CGG) in alum were measured (*n* = 3). The B1‐8^hi^ mice are genetically engineered to express BCRs with high affinity to NP [51], and also in this model, we observed a high frequency of NOX_drop_^+^ B cells on day 7 (44 ± 14%, Figure S11). This observation supported the hypothesis that BCR signaling also induced NOX activity.

### 2.4 NOX_drop_^+^ Plasma Cells Display Lower IgG Secretion Rates and an Enriched Fraction of Low IgG‐secreting Cells

After demonstrating a relationship between NOX expression and activity with BCR and TLR4 signaling, we investigated a possible relationship with functionality in PCs, that is, the expression and secretion of antibodies, respectively. First, we examined the *IGHG* and *IGHM* expression levels in NOX_mRNA_^−^ and NOX_mRNA_^+^ PCs. The *IGHG* expression level was similar over time (nonsignificant difference, Figure S12A), and generally seemed to be more upregulated in NOX_mRNA_^+^ PCs compared with NOX_mRNA_^−^ PCs. However, only day 3 showed a significant upregulation of *IGHG* in NOX_mRNA_^+^ PCs (*p*‐value 0.03). Additionally, changes in *IGHM* expression levels over time were not significantly different, although NOX_mRNA_^+^ *IGHM* ^+^ PCs tended to express always lower levels of *IGHM* compared with their NOX_mRNA_^−^ counterparts (Figure S12B). Next, we split the data into immature, mature, and apoptotic cells (Figure S12C–G). In line with the NOX_mRNA_^+^ PCs, the NOX_mRNA_^+^ immature, mature, and apoptotic PCs showed a higher *IGHG* expression level compared with their NOX_mRNA_^−^ counterparts except mature PCs on day 14 and apoptotic PC on day 7, where the expression levels were similar. Notably, *IGHG* reads were almost exclusively observed in NOX_mRNA_^+^ immature PCs on days 3 and 7, and NOX_mRNA_^+^ apoptotic PCs on day 7. The ratio of *IGHM* reads between the NOX_mRNA_^+^ and NOX_mRNA_^−^ PC subsets fluctuated over time without displaying a clear overall pattern.

After analyzing differences on the transcriptomic level, the relation between active NOXes with the IgG secretion rate was investigated. For this purpose, we additionally integrated our previously published bioassay to assess the IgG secretion rates to the droplet measurements (quantitative range between 9 and 285 IgG/s, see Figure S13) [38]. As the IgG probe labeled with A647 was used, the signal of the IgG probe was reduced if the cells displayed active NOXes. Due to the signal loss, we could not reliably detect and determine the IgG secretion rates of NOX_drop_^+^ cells. However, we measured the distribution of the IgG secretion rates of NOX_drop_^−^ IgG‐secreting cells (IgG‐SCs) and compared these with the distribution obtained from all IgG‐SCs when only using the IgG bioassay, that is, where no signal loss was observed. Figure 3C displays the IgG secretion rate distribution of all IgG‐SCs (grey) and NOX_drop_^−^ IgG‐SCs (yellow) for all analyzed days. On day 0, around half of all IgG‐SCs showed a secretion rate between 9 and 25 IgG/s, followed by a second population with secretion rates >200 IgG/s, and no IgG‐SCs displayed rates between 25 and 200 IgG/s. In general, NOX_drop_^−^ IgG‐SCs displayed a higher frequency of cells secreting ≥285 IgG/s over all days. Indeed, on day 7, all cells displaying a secretion rate of ≥285 IgG/s in the total IgG‐SCs were observed to be part of the NOX_drop_^−^ subpopulation, suggesting that NOX_drop_^+^ PCs were increased in the low‐secreting IgG‐SCs. However, no exclusive differentiation between low and high IgG‐SCs was possible depending on their NOX status, as both populations, although enriched, displayed both phenotypes.

### 2.5 The NOX^+^ Phenotype Correlates with Higher Modulation of Metabolic Pathways and Lactate Secretion Rates

Given that ROS can potentially reprogram cellular metabolism [38, 39], we next investigated a potential relationship between the NOX status and modulation of metabolic pathways in B cells. Therefore, different metabolic pathways were investigated during the immune response by analyzing DEGs (p‐value <0.05, see methods, or the definition of glycolysis, lactate metabolism, and TCA cycle in Bucheli et al. [38]). The differences in the metabolic pathways were determined by comparison with the pooled B cells from the different days, allowing conclusions about the pathways' modulation within the different subpopulations.

**FIGURE 4:**
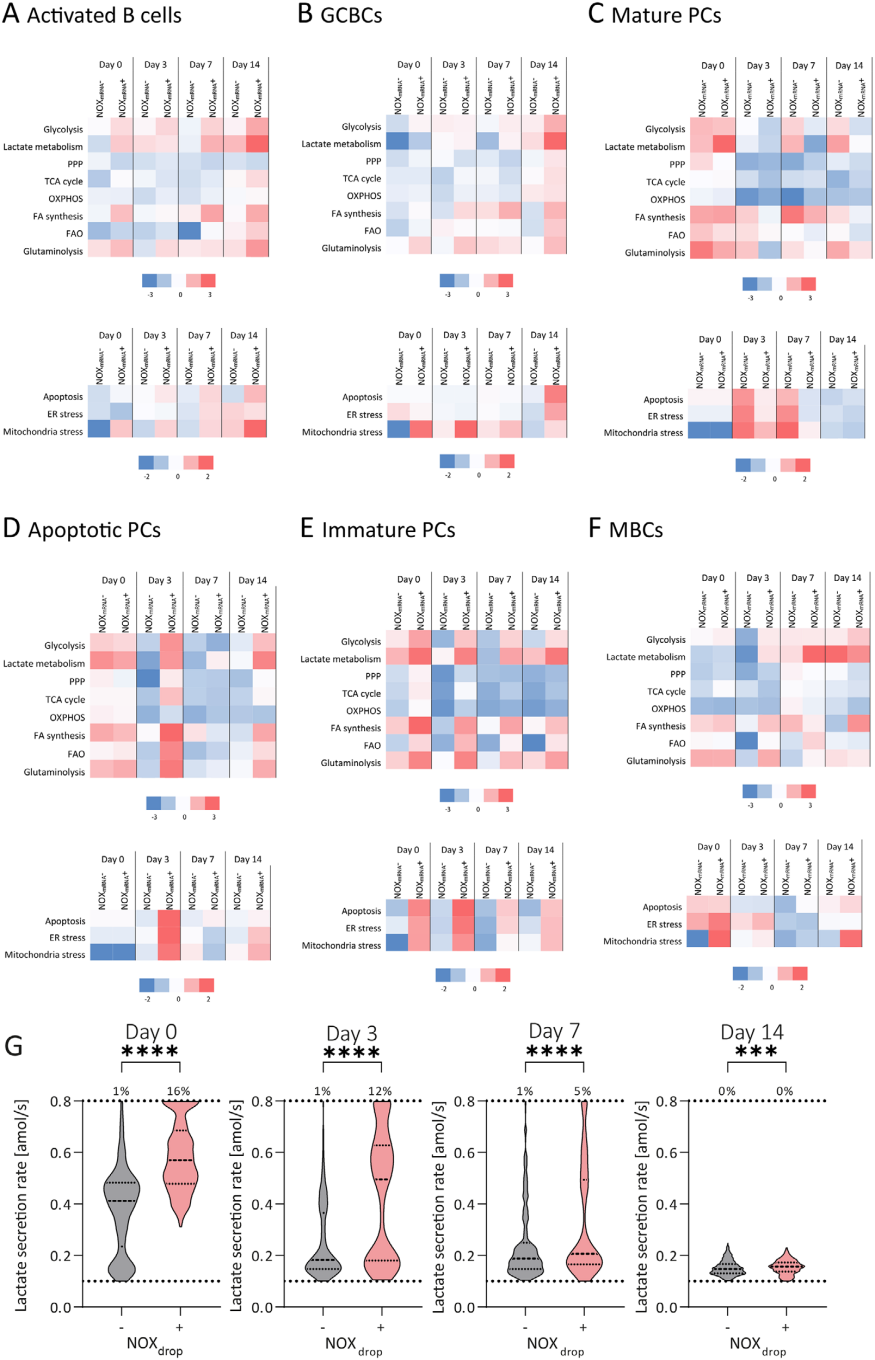
Top row: Expression level of genes associated with selected metabolic pathways in NOX_mRNA_^−^ (left column) and NOX_mRNA_^+^ (right column) (A) activated B cells, (B) GCBCs, for PC subpopulations divided into (C) mature PCs, (D) apoptotic PCs, and (E) immature PCs during the immune response, and (F) MBCs. As all plasmablasts were NOX_mRNA_^−^, this subset was excluded from the analysis. The log2 fold change of DEGs involved in each pathway was considered (*p*‐value <0.05). Bottom row: Heatmap representing the expression level of apoptosis, ER stress, and mitochondrial stress‐associated genes. (G) Distribution of lactate secretion rates of NOX_drop_^−^ and NOX_drop_^+^ cells during the secondary immune response. The dotted lines represent the borders of the quantitative range (0.1–0.8 amol/s [38]). The number above the distributions indicates the frequency of cells with a lactate secretion rate of ≥0.8 amol/s for the respective category. The level of statistical significance is denoted as ***p < 0.001 and ****p < 0.0001.

In activated B cells, genes associated with glutaminolysis appeared upregulated throughout the immune response (Figure 4A). In addition, glycolysis and fatty acid (FA) synthesis were positively modulated in the NOX_mRNA_^+^‐activated B cells. Lactate metabolism was dynamic in both subpopulations, whereby it remained positively modulated in NOX_mRNA_^+^ activated B cells throughout the immune response, whereas only on days 3 and 14 in NOX_mRNA_^−^ activated B cells. On day 14, the TCA cycle and fatty acid βoxidation (FAO) genes were also upregulated in NOX_mRNA_^+^‐activated B cells. In GCBCs, all metabolic pathways were negatively modulated on day 0 except for glutaminolysis and glycolysis in NOX_mRNA_^+^ GCBCs (Figure 4B). After the booster, NOX_mRNA_^+^ GCBCs also positively modulated lactate metabolism and FA synthesis. On day 14, all pathways were highly modulated in the GCBCs compared with the rest of the B cells, especially lactate metabolism. In contrast, most metabolic pathways remained negatively modulated at the transcriptional level in the NOX_mRNA_^−^ GCBCs. Mature PCs showed an upregulated expression of genes related to almost all metabolic pathways on day 0 for both the NOX_mRNA_^−^ and the NOX_mRNA_^+^ subpopulations (Figure 4C). Upregulation was observed over time in the NOX_mRNA_^−^ population whereas the NOX_mRNA_^+^ mature PCs showed decreased expression for almost all days and pathways when compared with all other B cells present in the sample. In apoptotic PCs (Figure 4D), the expression of all metabolic pathways‐related genes was upregulated in both subpopulations on day 0. On all analyzed days after the booster, the NOX_mRNA_^−^ apoptotic PCs showed negatively modulated metabolic pathways. On the contrary, the gene expression in NOX_mRNA_^+^ apoptotic PCs was highly dynamic, remaining upregulated on day 3 (all except OXPHOS), followed by a downregulation on day 7 and a second upregulation of selected pathways on day 14. For immature PCs (Figures 4E), a generally similar picture was observed as for apoptotic PCs, with a notable exception on day 7 (where NOX_mRNA_^+^ immature PCs upregulation remained high) and a more constant overexpression of genes involved in lactate metabolism, independent of NOX status. Comparing the different PC subpopulations reveals that mature PCs behave oppositely to apoptotic and immature PCs. While the NOX_mRNA_^+^ cells in apoptotic and immature PCs tended to show a positive modulation of metabolic pathways (mainly glycolysis, lactate metabolism, FA synthesis, FAO, and glutaminolysis), the NOX_mRNA_^−^ cells in the mature PCs did so. NOX_mRNA_^+^ MBCs showed primarily activated glycolysis, FA synthesis, and glutaminolysis during the immune response (Figure 4F). In addition, genes associated with lactate metabolism only were upregulated after the booster, especially on day 7. On day 7, FAO was also positively modulated. In contrast, NOX_mRNA_^−^ MBCs displayed negatively modulated metabolic pathways on day 3 that became positively modulated on day 7, except for FAO and glutaminolysis.

The relationship between lactate metabolism and NOX^+^ phenotype was further investigated using droplet measurements. Cells were divided into NOX_drop_^−^ and NOX_drop_^+^ based on detecting active NOXes, and the distributions of lactate secretion rates of the two groups were compared on days 0–14 (Figure 4G). The distributions differed significantly on all days, with the NOX_drop_^+^ subpopulations having a higher median secretion rate (*p*‐value <0.0001 on days 0, 3, and 7, *p*‐value 0.0004 on day 14). In addition, cells with high lactate secretion rates (≥0.8 amol/s) accumulated in the NOX_drop_^+^ subpopulation. Overall, the population of cells with secretion rates >0.4 amol/s was more pronounced in the NOX_drop_^+^ subpopulation. Accordingly, there seems to be a link between the active state of NOXes and lactate metabolism.

### 2.6 Genes Associated with Apoptosis and Stress are Upregulated in Cells Expressing NOXes

Finally, we investigated a relationship between NOX expression and cellular stress responses. Therefore, apoptosis, the endoplasmic reticulum (ER), and mitochondrial stress responses were analyzed based on the expression level of genes (see methods, Table S2). In activated B cells, NOX_mRNA_^+^ cells displayed increased expression of apoptotic markers after the booster over the first 14 days (Figure 4A). In addition, ER stress was positively modulated in NOX_mRNA_^+^‐activated B cells on days 7 and 14, and in NOX_mRNA_^−^‐activated B cells on day 14. Mitochondrial stress was positively modulated in NOX_mRNA_^+^ activated B cells on all days, whereas in NOX_mRNA_^−^ cells only on day 14. In NOX_mRNA_^+^ GCBCs, apoptosis and ER stress were positively modulated only on day 14, whereas mitochondrial stress responses were positively modulated throughout the immune response (Figure 4B). NOX_mRNA_^−^ GCBCs showed downregulation of genes related to ER stress on day 0 and mitochondrial stress on days 3 and 7. In addition, NOX_mRNA_^+^ MBCs exhibited positively modulated mitochondrial stress on day 14. In NOX_mRNA_^+^ mature PCs, apoptosis, ER, and mitochondrial stress were downregulated when compared with their NOX_mRNA_^−^ counterparts early after re‐immunization (days 3 and 7, Figure 4C). Conversely, NOX_mRNA_^+^ apoptotic PCs displayed highly upregulated apoptosis, ER, and mitochondrial stress on day 3 and ER and mitochondrial stress on day 14 (Figure 4D). Immature PCs showed increased stress and apoptosis on all days in the NOX_mRNA_^+^ cells (Figure 4E). As already observed in the study of metabolic pathways, the PC subsets also appear to differ here, with the NOX_mRNA_^+^ cells of the apoptotic and immature PCs exhibiting more upregulated apoptosis and stress, whereas in the mature PC, the NOX_mRNA_^−^ cells did. As no NOX_mRNA_^+^ plasmablasts were found in the data set, a comparison based on NOX status was not possible. Lastly, genes related to apoptosis were upregulated on day 14 in both MBC subpopulations but to a greater extent in NOX_mRNA_^+^ (Figure 4F).

## 3 Discussion

This study confirmed the presence of NOX enzymes upon B‐cell activation and introduced a novel method to assess the activity of NOX enzymes. This method is advantageous compared with traditional studies focusing on the expression, as it allows the direct detection of activated NOX enzymes and the study of NOX^+^ cells at the single‐cell level. Especially the latter was important to perform correlation studies of NOX activity and, for example, cellular functionalities like antibody secretion rates.

The primary role of NOXes during immune responses has been described as the production of ROS [20, 28, 29], which have been shown to act and modify intracellular signaling pathways as secondary messengers [22, 23, 25, 26, 52]. Taking previous studies applying NOX inhibitors and using knockout mice as a starting point [21, 24, 27, 31, 32, 33], our study aimed to detect expression levels and functionally active NOXes in splenic B cells over the time course of an immune response and to study the relationship between NOXes and BCR and TLR4 signaling, cellular function, metabolism, stress markers, and apoptosis. Our transcriptomic analysis revealed that the genes encoding the NOX complex isoforms NOX1 and NOX2 were differentially expressed throughout the first 14 days of the secondary immune response, and we did not detect any expression of NOX3 and ‐4 and DUOX1 and ‐2. This finding contrasts a previous report indicating the expression of NOX1‐3 and DUOX2 in activated B cells [24]. These differences may be due to variations in the model system: we extracted splenic B cells from immunized BALB/c mice, whereas Feng et al. [24] stimulated splenic B cells from C57BL/6 mice ex vivo using anti‐IgM antibodies.

Besides the upregulated expression of NOX1 and NOX2 in activated B cells, we could also observe their upregulation in additional B‐cell subsets, namely GCBCs, PCs, and MBCs. Notably, GCBCs from the LZ were found to contribute significantly to the population of NOX_mRNA_^+^ B cells on day 0, that is 28 days after primary immunization, indicating a potential role of NOXes in the extended GC response and aligning with the antigen binding during the positive selection within the GCLZ. This observation is consistent with a report showing activated NOX enzymes in GCBCs using NADPH FLIM [53]. Moreover, we also found that NOX_mRNA_^+^ GCBCs were in the majority carrying GCLZ markers after secondary immunization (i.e., on days 3, 7, and 14). Lastly, based on the high proportion of NOX_mRNA_^+^ PCs and MBCs on day 0, we hypothesized that NOX_mRNA_^+^ GCBCs gave rise to these cell populations. Thereby, the appearance of plasmablasts and immature PCs as NOX_mRNA_^−^ is hypothesized to be potentially due to gene expression reprogramming as these cells shift their focus from proliferation and mutation to preparing for antibody production, leading to a temporary downregulation of NOX expression compared with all B cells.

Although we did not observe a correlation between NOX expression (scRNA‐Seq) and activity (in droplets), the droplet data supports that more than one B‐cell subset displayed NOX activity as the frequency of NOX_drop_^+^ cells was higher than the observed frequency of IgG‐SCs. Moreover, it highlights the importance of complementing transcriptomic assessments of NOXes with direct functional measurements. As in‐droplet staining for intracellular targets could not be combined with functional analysis, we were not able to pinpoint the subpopulation(s) that displayed activity in our assay. Furthermore, we did not sort the cells prior to droplet analysis as sorting was shown to impact functionalities [54, 55]. Therefore, we compared the characteristics of all NOX_drop_^+^ cells or NOX_drop_^+^ IgG‐SCs with the NOX_mRNA_^+^ subsets. Further development of system(s) to close the gap between transcriptomic assessments and direct functional measurements is required, for example, a system to select droplets containing cells of certain characteristics followed by sequencing.

Importantly, we also did not distinguish between antigen‐specific cellular subsets and bystander subsets in both methods. Observed differences between expression and direct functional measurements could also be, at least in part, due to the dynamics and localization of immune cells within the organ over the assayed time points. The microenvironment might alter the cellular phenotypes presented in this paper, especially on the level of metabolic preferences and regulation. However, interestingly, these changes seemed not restricted to a specific subpopulation, indicating that, while there most certainly is an influence of the microenvironment, there were cell‐specific differences at play.

Using the newly introduced droplet method to detect active NOXes, we investigated the frequency of cells with active NOX complexes throughout the first 14 days of the immune response following re‐immunization. The frequency of active NOXes appeared to vary over time, with an intermediate peak on day 3 and an absolute maximum on day 14 when the TLR4 agonist MPLA was employed, suggesting that TLR4 signaling played a particular role in NOX activity in the early activation phase of B cells and during the GC response. In contrast, the sole activation of BCR signaling by TT/alum resulted in a much more stable frequency of cells with active NOXes. Across all B‐cell subsets, BCR and TLR4 signaling pathways were more positively modulated in the NOX_mRNA_^+^ B cells than their counterparts, indicating a link between both BCR and TLR4 signaling and the upregulation of NOXes. The observed link aligned with literature describing ROS as a second messenger in BCR and TLR4 signaling [20]. Of further interest was the duration during which the expression of TLR4 signaling‐associated genes was upregulated. On day 14 and 0, that is, 28 days after primary immunization, the genes were still upregulated, although, based on the half‐life of the alum depot and LPS (of which MPLA is a derivative) [56, 57, 58], we did not expect MPLA to still be present in relevant concentrations. The observed TLR4 modulation may be due to damage‐associated molecular patterns (DAMPs), which include molecules released by damaged or stressed cells [59]. Accordingly, we hypothesized that the observed NOX activity during the GC reaction was due to positive selection, in line with NOXes being upregulated in the LZ, and the associated BCR ligation and (potentially) the TLR4 signaling by DAMPs. Our explanation for the higher frequency of NOX_drop_^+^ cells in the TT/AS04 condition, when compared with TT/alum, was an increased activation and formation of GCs in response to TT/AS04, and activation by MPLA that we were also able to recapitulate in in vitro stimulations. Another possibility could be that other receptors besides BCR and TLR4 were involved, particularly cytokine receptors that could be linked to ROS production [20]. Indeed, different immune responses have been reported for AS04 and alum, leading to different secreted cytokine repertoires [57, 60, 61, 62, 63]. Thus, the difference in NOX_drop_^+^ frequency could also result from these different cytokine repertoires, either directly or indirectly acting on the B cells.

After having demonstrated this association of NOX enzymes and BCR and TLR4 signaling, we aimed to investigate the impact of NOXes on cellular functionalities. In line with the literature describing that treatment with NAC or the knockout of NOX2 resulted in higher antibody productions [24, 41, 42, 43, 44, 64], we observed the trend that NOX_drop_^−^ IgG‐SCs had higher secretion rates compared with all IgG‐SCs on almost all days, and high‐secreting IgG‐B cells were enriched in the NOX_drop_^−^ subset. Albeit NOX_mRNA_^+^ PCs seemed to express more *IGHG* (only significantly different on day 3), our additional transcriptomic analysis of ER stress could partially explain this discrepancy between expression and secretion. The unfolded protein response and the added stress could explain why the cells exhibited high *IGHG* expression levels but low IgG secretion rates, but additional experiments are needed to decipher these molecular pathways and interactions. By further transcriptomic analysis of the PC subsets, we were able to link ER stress and increased *IGHG* reads to NOX_mRNA_^+^ apoptotic, immature, and mature PCs early after the secondary immunization, further supporting this hypothesis. However, this hypothesis does not explain the upregulated ER stress in NOX_mRNA_^−^ mature PCs, which was indeed even stronger than in NOX_mRNA_^+^ mature PCs. However, as the sum of NOX_mRNA_^−^ immature and apoptotic PCs and plasmablasts exceeds the number of NOX_mRNA_^−^ mature PCs, their IgG secretion rates could have been masked in the IgG distribution. As mentioned previously, a limitation of the droplet assay is the lack of differentiating IgG‐SCs into plasmablasts and PC subsets. Nevertheless, NOX activity seems to be at least partly linked to antibody secretion, as NOX activity correlated with lower antibody secretion rates (Figure 3C).

Considering the relationships between B‐cell activation and metabolism [34, 35, 36, 65, 66], we aimed to investigate a link between NOX enzymes and metabolic pathways in greater detail. Transcriptomic analysis showed that the metabolic profile of NOX_mRNA_^−^ and NOX_mRNA_^+^ B cells of all subpopulations varied over time of the immune response. Overall, the modulation of selected metabolic pathways was higher in NOX_mRNA_^+^ cells compared with NOX_mRNA_^−^ cells, with the exception of mature PCs. This observation supported the hypothesis that NOX activity is crucial for B‐cell activation and thus linked to metabolic changes to meet the enhanced energy and nutrient demands. As a balanced ratio of NADP^+^/NADPH plays an important role in various metabolic pathways, we expected correlations between the expression of NOXes and activation of the pentose phosphate pathway (PPP) (whereby NADP^+^ is reduced to NADPH [67]) as well as the inactivation of FA synthesis (whereby NADPH is oxidized to NADP^+^ [68]). Surprisingly, no such correlation was observed. An unbalanced ratio of NADP^+^/NADPH can result in a reduced antioxidant capacity, as NADPH serves as a cofactor [69]. Indeed, our transcriptomic analysis revealed positively modulated mitochondrial stress responses in NOX_mRNA_^+^‐activated B cells, GCBCs, MBCs, and apoptotic and immature PCs. However, the NOX_mRNA_^+^ B cells also exhibited positively modulated glutaminolysis, which was shown to produce glutathione [70]. Glutathione has antioxidant properties [71], potentially reducing the damaging effects of increased ROS production. One metabolic pathway consistently more positively modulated in NOX_mRNA_^+^ B cells was lactate metabolism, an observation we could also confirm by assessing the cellular lactate secretion rate. Indeed, the distributions of lactate secretion rates of the NOX_drop_^−^ and NOX_drop_^+^ subpopulations differed significantly on all measurement days, with the NOX_drop_^+^ cells displaying higher lactate secretion rates. We excluded an influence of the measurement of NOX activity on detecting high secretion rates and vice versa, as both reactions use NADPH and are, therefore, in competition. Accordingly, an underestimation of the lactate secretion rate or NOX enzyme activity might rather occur. Therefore, our results link the reports that B‐cell activation leads to increased glycolysis [34, 35, 36, 38] and the activation of NOX enzymes [20, 24, 31, 72].

NOXes can cause oxidative stress within the ER and mitochondria not only by disrupting the cellular redox balance through the consumption of NADPH but also through the extensive production of ROS. Indeed, ER and mitochondrial stress modulations were primarily detected in the NOX_mRNA_^+^ subpopulations of the B‐cell subsets. In particular, mitochondrial stress was positively modulated in NOX_mRNA_^+^ activated B cells, GCBCs, and PCs over the monitored period of the immune response with the exception of the mature PC subset. Mitochondrial stress could be caused not only by the ROS produced by NADPH oxidases but also by the ROS produced directly by the mitochondria [31]. Nevertheless, we observed that NOX_mRNA_^+^ B cells upregulated genes involved in apoptosis more than their counterpart, potentially due to the negative effects of enhanced ROS production, again with the notable exception of mature PCs. At the same time, elevated production of ROS could also cause excessive inhibition of phosphatases, resulting in overactive BCR signaling and thus causing cell death, as shown by Yam‐Puc and colleagues [9]. In our assessment, mature PCs exhibited distinct characteristics compared with the other B cell subsets, with NOX_mRNA_^+^ PCs displaying on average decreased expression levels of genes associated with apoptosis, ER, and mitochondrial stress. Indeed, cells with high protein expression require an increased antioxidant defense system to protect from ROS‐induced apoptosis [73]. Accordingly, we hypothesize that the observed NOX_mRNA_^+^ mature PCs developed system(s) to counterbalance ROS‐induced stress.

Interestingly though, when looking at the mature PC subpopulation in the BM, the frequency of NOX_mRNA_^+^ mature PCs decreased over time, indicating a shift toward NOX downregulation once the cells relocated to the BM. Therefore, these results could indicate that NOX expression (upregulation) is a potential short‐term marker in mature PCs for their fate and would indicate that NOX_mRNA_^+^ in mature PCs might serve as a predictive marker for longevity for freshly generated mature PCs in the spleen. Yet, additional, more focused studies surrounding this question are needed, and additional studies focusing on the influence of ROS on the microenvironment and surrounding cells will provide insights into these interactions.

To conclude, our study demonstrated that NOX activity influences B‐cell activation and provides insight into the role of NOX and ROS as potential mediators in processes related to B‐cell differentiation, functionality, and survival. However, further studies are required to investigate and validate the underlying mechanism of these correlations.

## 4 Methods

### 4.1 Immunization of Mice

BALB/cJRj (Janvier Labs, female, 7–10 weeks old at time of primary immunization) were immunized with recombinant tetanus toxin heavy chain fragment C (TT, 10 µg, FinaBio). TT was added to physiological water (InvivoGen, vac‐phy) and supplemented with adjuvant system 04 (AS04, 5 µg MPLA‐SM, tlrl‐mpla2, and alhydrogel adjuvant 2%, vac‐alu‐250, both InvivoGen) or alum alone in a ratio of 1:1 (*v/v*). The mice were re‐immunized four weeks after the primary immunization with the same mixture. The immunizations were administered intraperitoneally (i.p.) with 100 µL volume. The immune response was examined 4 weeks after primary immunization (called day 0) or on days 1, 2, 3, 4, 7, and 14 after re‐immunization. Experiments were performed according to institutional guidelines and Swiss Federal regulations and were approved by the cantonal veterinary office Zurich (animal experimentation permission ZH215/19).

In addition, spleen samples from B1‐8^hi^YellowCaB mice (one female mouse, 7 months old at the time of primary immunization, and two male mice, 6 months old at the time of primary immunization) were provided by Prof. A. E. Hauser's group (Charité). These mice were immunized i.p. with 4‐hydroxy‐3‐nitrophenylacetyl‐chicken gamma globulin (NP‐CGG, 100 µg) supplemented in alum three times with a time interval of 21 days. The immune response was studied 5–7 days after the third immunization.

### 4.2 Cell Isolation from Spleen

The splenic cells were isolated as described elsewhere [38, 74]. In short, the spleens were dissociated by gently passing the tissue through a 40 µm cell strainer. Red blood cell lysis was performed using BD Pharm lyse Lysing buffer (1 min at room temperature), followed by filtration (40 µm cell strainer), and two washing steps with MACS buffer (D‐PBS, #P4417, with added 2 mM EDTA, #03620, and 0.5% (*w/v*) BSA, #A3059, all Sigma Aldrich). Cells from the B‐cell lineage were isolated using the Pan B Cell Isolation Kit II (Miltenyi Biotec) according to the manufacturer's protocol, resulting in a purity of 95 ± 2% [38]. Cells were stored on ice (≤12 h).

### 4.3 In Vitro Cell Stimulation

A part of the splenic cells from day 0 was ex vivo stimulated with MPLA (1 µg/1 × 10^6^ cells/mL) for 4 h at 37°C (these data, differently analyzed, are also part of the literature [38]).

### 4.4 Droplet‐based Measurements

#### 4.4.1 Aqueous Phase I: Preparation of Cells

The cells were stained with CellTrace Violet (5 µM, Thermo Fisher, C34571) for 30 min on ice. After a washing step with MACS buffer, the cells were collected (400 g, 5 min, 4°C) and resuspended at a density of 0.8–2 × 10^6^ cells/100 µL (*λ* = 0.2–0.5) in assay buffer (RPMI 1640 without phenol red, #11835063, 10% (*v/v*) KnockOut Serum Replacement, #10828010, 1X penicillin‐streptomycin, #10378016, 25 mM MOPS pH 7.5, #J61843, 0.1% (*v/v*) Pluronic F‐127, #11835030, all Thermo Fisher, and 0.5% (*w/v*) recombinant human serum albumin, A9731, Sigma Aldrich). If indicated, *N*‐acetylcysteine (NAC, Thermo Fisher, C10492) was added to the cell staining solution (10 mM) and aqueous phase I (20 mM) on day 7.

#### 4.4.2 Aqueous Phase II: Preparation of Nanoparticles and Detection Reagents

Paramagnetic nanoparticles (Strep Plus, 300 nm, Ademtech) for measurement of IgG secretion were prepared as described before [38, 74, 75]. The IgG assay contained a goat anti‐mouse IgG Fc antibody (SouthernBiotech, 1033‐30, labeled with Alexa Fluor 647) at a final, in‐droplet concentration of 75 nM. In the experiments with NAC, the IgG probe was replaced by Alexa Fluor 647 NHS Ester (Thermo Fisher, A37573), which was resuspended in D‐PBS, stored for >2 weeks in the fridge, and added at a final, in‐droplet concentration of 150 nM. The commercial fluorometric lactate assay (Lactate Assay Kit, Sigma‐Aldrich, MAK064) was used as described in Bucheli et al. [38], that is, 8 µL enzyme mix and 0.5 µL fluorescent probes were added per 100 µL aqueous phase II. CellROX Green Reagent (Thermo Fisher, C10492) was added in a final, in‐droplet dilution of 1:800 of the manufacturer's stock solution as previously published [38]. The aqueous phase II solution was stored on ice (≤12 h) and resuspended before measurements.

#### 4.4.3 Microfluidic Droplet Generator and Observation Chamber Assembly

As previously published, the PDMS chip for water‐in‐oil emulsion droplet generation and the 2D observation chamber were prepared [74, 75].

#### 4.4.4 Data Acquisition and Analysis

According to previous publications, droplets with a volume of 50 pL were generated and transferred into the 2D observation chamber [74, 75]. During the droplet generation, the aqueous phase I was cooled using ice‐cold water. The observation chamber was closed and, for the analysis of splenic cells from BALB/c mice, mounted onto an inverted fluorescence microscope for imaging (Ti2 Eclipse, Nikon) equipped with a motorized stage, excitation light (Lumencor Spectra X), 10x objective (NA 0.45, Nikon), and a digital CMOS camera (ORCA‐Fusion C14440, Hamamatsu, in 2×2 binning mode). For the measurements of the splenic B cells of B1‐8^hi^YellowCaB mice, the chamber was mounted onto an inverted fluorescence microscope (Ti2 Eclipse, Nikon, AMBIO Facility at Charité—Universitätsmedizin Berlin) with a motorized stage, excitation light (Lumencor Spectra X), 10× objective (NA 0.3, Nikon), and a digital sCMOS camera (PCO.edge, in 2 × 2 binning mode). Using appropriate bandpass filters (DAPI, FITC, TRITC, and Cy5 filter set, Semrock), fluorescence signals were recorded at 37°C under ambient oxygen and carbon dioxide levels. An array of 10×10 images was acquired, covering around 50,000 droplets. For the measurements of BALB/c mice‐derived cells, a time series was recorded using a 10 min interval over a total duration of 50 min, resulting in six measurements per experiment. For the B1‐8^hi^YellowCaB mice‐derived cells, a 12 min interval over a total duration of 48 min was applied, resulting in five measurements per experiment.

The acquired images were analyzed using an updated custom Matlab script (previous versions published in [68, 69]). The extracted values were exported to Excel (Microsoft) to analyze functional and metabolic parameters as described below. The Kolmogorov‐Smirnov test was used to compare distributions and transcriptomic analysis, and Welch's *t*‐tests were used to compare two groups (GraphPad Prism, Version 8.2.0). Where more than two groups were compared, a Brown‐Forsythe and Welch ANOVA test was used. The statistical significance levels were indicated as *p < 0.05, **p < 0.01, ***p < 0.001, and ****p < 0.0001.

#### 4.4.5 Assessment of NOX Activity

The fluorophore Alexa Fluor 647 (A647), either as free dye or coupled to the IgG probe, was combined with the lactate assay, and the loss of the fluorescence signal intensity of A647 of the whole droplet was used as an indicator for the presence of active NOXes. Based on the fluorescence signal of droplets containing only the IgG probe labeled with A647, the threshold for identifying NOX_drop_^+^ cells has been defined as follows:

$$\mathrm{Threshold}=\mathrm{median}\left(x_{n}\right)+3\;SD\left(x_{n}\right),$$

$$\;x_{n}=\frac{100\%}{\mathrm{signa}l_{n\left(t1\right)}}\;\;\cdot \left(\mathrm{signa}l_{n\left(t1\right)}-\mathrm{signa}l_{n\left(\mathrm{lowest}\right)}\right),$$

where *x*_n_ was calculated for every individual droplet containing a cell and signal_n(t1)_ represented the droplet's fluorescence intensity at the first time point, whereas signal_n(lowest)_ was its lowest fluorescence intensity between the second and last time points. Using the indicated formulas, the threshold was set at 10.3%. Droplets with a signal loss relative to their first signal greater than 10.3% were classified as NOX_drop_^+^. Across all measurements used in this study, which included only the IgG probe and not its combination with the lactate assay, an average of 0.4 ± 0.6% cells (*n* = 14) exceeded this threshold. The reasons were erroneous droplet tracking as well as inaccurate stitching of the images.

#### 4.4.6 Assessment of IgG Secretion Rates

The calibration curve was fitted and the IgG secretion rates were calculated according to the literature [38]. In brief, the employed sandwich immunoassay resulted in the relocation of the IgG detection probe to the functionalized nanoparticles upon IgG secretion. The ratio between the mean signal on the nanoparticles and the mean signal of the whole droplet resulted in fluorescence relocation. The fluorescence relocation signal was calibrated using different IgG reagents (mouse anti‐human IgG4 Fc IgG1, Invitrogen, A‐10651; mouse anti‐human TNF‐α IgG1, eBioscience, 14‐7348‐85; mouse anti‐human CD3 IgG2a, PeproTech, 05111–20; mouse anti‐human CD20 IgG2b, PeproTech, 02311–20). The resulting curves were fitted using an exponential model, and the quantitative range spanned from 9 to 258 IgG/s. The fluorescence relocations obtained during cell measurements were converted to IgG concentrations using the calibration curve. Droplets fulfilling the criteria stated in the literature were visually verified, and subsequently, the cellular secretion rates (IgG/s) were derived by dividing the IgG increase between time points by the time interval and calculating the cell's average secretion rate [38]. Cells with a secretion rate ≥9 IgG/s (i.e., equal or above the assay's LoD) were defined as IgG‐SCs.

#### 4.4.7 Assessment of Lactate Secretion Rates

The lactate secretion rates were calculated according to Bucheli et al. [38], whereby the droplet's fluorescence intensity was determined and the intensity converted into a lactate concentration, followed by calculating the cell's average lactate secretion rate (quantitative range 0.1–0.8 amol/s). Droplets containing more than one cell were excluded.

#### 4.4.8 Assessment of Cellular ROS Production Level

Data from our previous study were re‐analyzed to study this question [38]. In short, the cellular ROS production level was assessed using a cell‐permeable, ROS‐sensitive dye. Upon ROS‐mediated oxidation, the dye bound to the DNA resulting in an increased cell signal over time. The signal increase [a.u./10 min] served as an indicator of the level of ROS production and was calculated until the cell reached its maximum cell signal, followed by extracting the average value.

### 4.5 scRNA‐Seq

The transcriptomic data from the ArrayExpress repository (https://www.ebi.ac.uk/biostudies/arrayexpress) with accession number E‐MTAB‐13205, primarily published in reference [38], were re‐analyzed in this study.

First, the different B‐cell subsets in the spleen and bone marrow (BM) were identified based on key surface and intracellular markers expressed throughout B‐cell development in mice (Table S1). Within each B‐cell population, we identified NOX_mRNA_^−^ and NOX_mRNA_^+^ subpopulations based on their expression levels of NOX isoforms NOX1‐4 and DUOX1‐2, NOX gene regulators *NOXO1* and *NOXA1*, and NOX complexes encoding genes *CYBA*, *CYBB*, *NCF1/2/4*, and *DUOXA1/2*. B cells that presented expression levels of those genes of ≤0 (log2 FC) were classified as NOX_mRNA_^−^ cells and >0 as NOX_mRNA_^+^ cells.

In addition, the expression of GC markers for the DZ and LZ and PC markers for apoptotic, immature, and mature PC as well as plasmablasts within the NOX_mRNA_^−^ and NOX_mRNA_^+^ cells were examined. We identified cells from the DZ and LZ within the GCBCs using the gene markers described in Table S1. Likewise, within the PC population, we distinguished apoptotic, immature, and mature PC as well as plasmablasts (Table S1) as well as the IgG‐expressing cells (IgG‐ECs), that is, PCs that expressed *IGHG* (all isotypes). The expression levels of *IGHG* of NOX_mRNA_^−^ and NOX_mRNA_^+^ PCs were studied throughout the immune response.

The BCR and TLR4 signaling pathways in NOX_mRNA_^−^ and NOX_mRNA_^+^ cells were analyzed. The software iDEP 0.96 [76] (available here: http://149.165.154.220/idep) was accessed to visualize BCR and TLR signaling changes throughout the immune response.

After identifying a selection of important metabolic pathways and their respective (*p*‐value <0.05) and expression levels (based on log2 fold change), their activities in NOX_mRNA_^−^ and NOX_mRNA_^+^ subpopulations were assessed. The used definition of glycolysis, lactate metabolism (generation), and TCA cycle can be found in Bucheli et al. [38]. In short, glycolysis starts from glucose being metabolized into glucose‐6‐phosphate and continues until the generation of pyruvate. Lactate metabolism considers the generation of lactate from pyruvate. Lastly, the TCA cycle starts with the formation of acetyl‐CoA. By doing so, we could distinguish whether B cells preferred the pathway that leads to lactate or to fuel the TCA cycle. The rest of the metabolic pathways, namely PPP, OXPHOS, fatty acid (FA) synthesis, FAβ‐oxidation (FAO), and glutaminolysis were based on the KEGG database.

The activity of apoptosis and gene markers associated with endoplasmic reticulum (ER) and mitochondrial stress in NOX_mRNA_^−^ and NOX_mRNA_^+^ populations were investigated (apoptosis: KEGG database, ER and mitochondrial stress: Table S2). Housekeeping genes, that is, genes that are constitutively expressed, specifically *ATF1/2/4/6/7, BTF3, E2F4, JUND, HPRT*, and *RPL3*, were used as internal controls to check for the potential bias in the RNAseq experiments and the resulting gene expression analysis using fold changes. As expected, the expression of these genes did not significantly alter in their log2fold changes over time (results not shown). Heatmaps and graphs were generated using R [77], Microsoft Excel, and GraphPad Prism Version 9.5.1.

## Author Contributions

Olivia T. M. Bucheli planned all experiments, performed and analyzed the droplet experiments, and drafted the manuscript. Daniela Rodrigues performed and analyzed the scRNA‐Seq experiments. Carolin Ulbricht and Anja E. Hauser contributed to the work with the B1‐8^hi^YellowCaB mouse samples. Klaus Eyer supervised the project. All authors commented on and revised the manuscript.

## Conflicts of Interest

The authors declare no conflicts of interest.

## Supporting information



Supporting Information

## Data Availability

The transcriptomic data generated and analyzed during the current study are available on the ArrayExpress repository (https://www.ebi.ac.uk/biostudies/arrayexpress) with accession number E‐MTAB‐13205. Further data that support the findings of this study are available from the corresponding author upon reasonable request.
