# Supplementary material for: Dynamic Activation of NADPH Oxidases in Immune Responses Modulates Differentiation, Function, and Lifespan of Plasma Cells
Source: Eur J Immunol. 2025 Feb 11;55(2):e202350975. doi: 10.1002/eji.202350975 (PMC11811814; doi:10.1002/eji.202350975)
Supplement: Supplementary file 1 — Supporting Information [file EJI-55-e202350975-s001.pdf]

Supporting Information

SITable 1: Key surface and intracellular gene markers that were applied to identify subpopulations of interest in spleen and bone marrow.

**Immature B cells**	_CD19, CD24, CD93, PTPRC (B220), PAX5, EBF1, IGHM_
**Naïve B cells**	_CD19, MS4A1 (CD20), CD27, CD80, PAX5, IgHD_<br>_or_<br>_CD19, CD37, MS4A1 (CD20), CCR6, BCL6, IgHD_
**Activated B cells**	_CD19, MS4A1 (CD20), CD27, CD80, PAX5, IGHG and/or IGHM, no IgHD_
**Memory B cells**	_CD19, CR2 (CD21), NT5E (CD73), POU2AF1 (OBF1), SPI-B_
**Germinal center B cells**	_CD19, CD37, MS4A1 (CD20), CCR6, BCL6, no IgHD_
**GC Dark Zone cells**	_Germinal center B cell markers +_<br>_CXCR4 (upregulated), FOXO1, CD83_$^{++}$*, _CD86_$^{++}$*
**GC Light Zone cells**	_Germinal center B cell markers +_<br>_CXCR4 (downregulated), HIF1a, cMYC, CD83_$^{+++}$*, _CD86_$^{+++}$*
**Plasma cells**	_SDC1 (CD138), CXCR4, PRDM1 (BLIMP1), XBP1_
**Plasmablasts**	_PC markers +_<br>_MYC, Ki67, PTPRC_
**Immature PCs**	_PC markers +_<br>_PRDM1_$^{+}$, _CXCR3_$^{+}$, _SLAMF6_$^{+}$, _CD93_$^{-}$
**Mature PCs**	_PC markers +_<br>_CXCR4_$^{+}$, _CD93_$^{+,}$ _PRDM1_$^{+++}$*, _EPCAM_$^{+}$, _FCER1G_$^{+}$, _CXCR3_$^{-}$, _SLAMF6_$^{-}$
**Apoptotic PCs**	_PC markers +_<br>_CASP3/7/9_$^{+}$

* _PRDM1_$^{+}$ means genes with log2 fold change between 0 and 1; _PRDM1_$^{+++}$ means genes with log2 fold change >1.

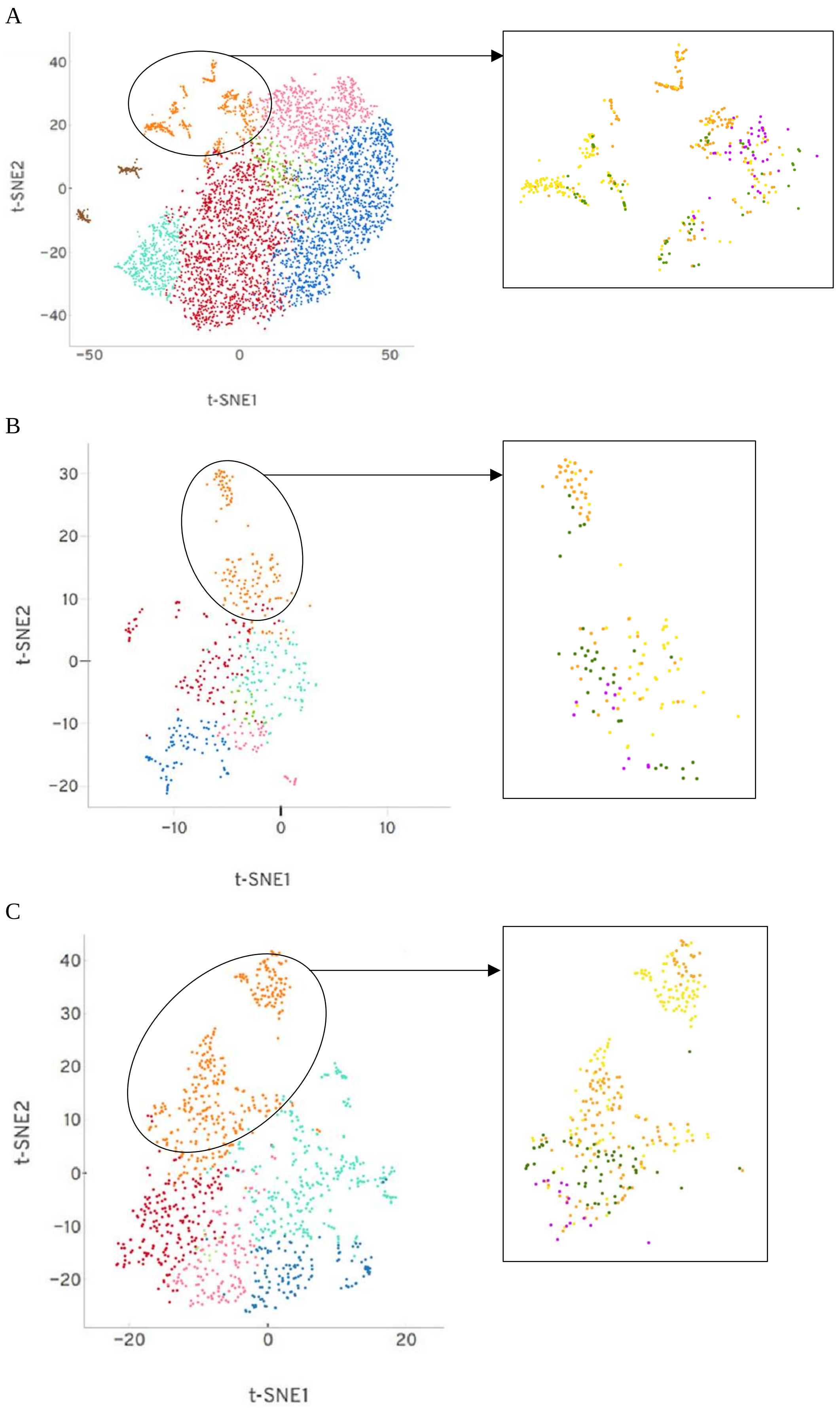
A
t-SNE2
t-SNE1
40
20
0
-20
-40
-50
0
50
B
t-SNE2
t-SNE1
30
20
10
0
-10
-20
-10
0
10
C
t-SNE2
t-SNE1
40
30
20
10
0
-10
-20
-20
0
20

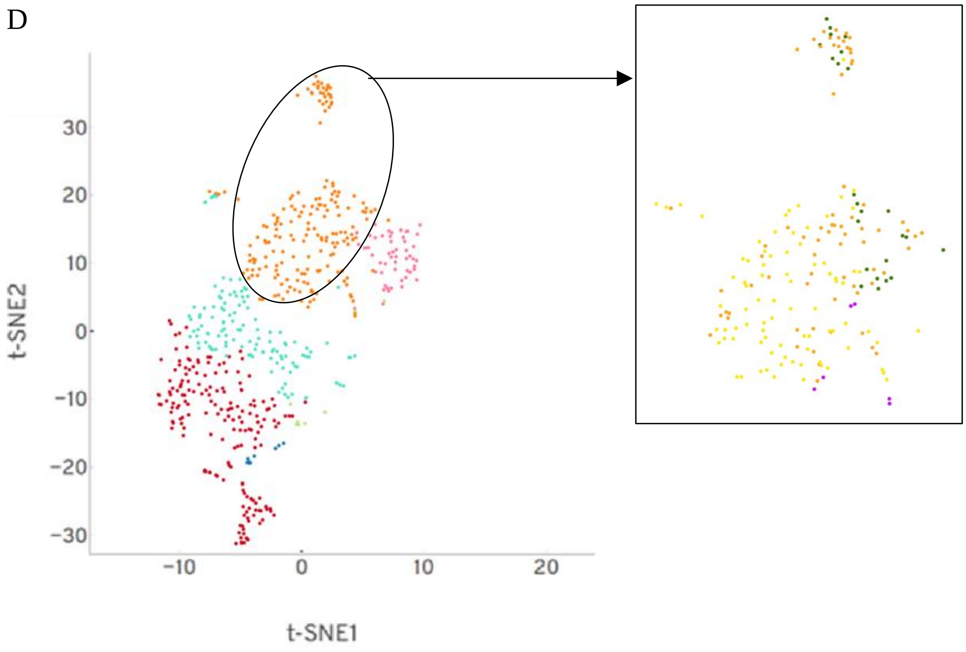

t-SNE plot:

■ Immature B cells ■ GC B cells ■ Activated B cells ■ Naïve B cells ■ PCs ■ Memory B cells ■ Others

In insert focused on PCs:

■ Immature PCs ■ Mature PCs ■ Apoptotic PCs ■ Plasmablasts

SIFigure 1: Identification and t-SNE projections (Loupe Browser) of the different cell populations in the spleen on (a) day 0, (b) day 3, (c) day 7 and (d) day 14.

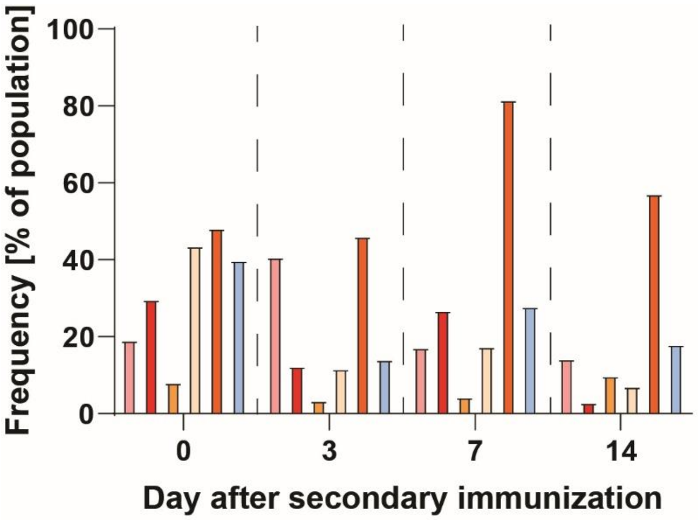

SIFigure 2: Frequency of NOX_{mRNA}^{+} B cells. Data from Figure 1D is represented as a percentage of the respective subpopulation. Activated B cells are displayed in pink, GC BCs in red, immature PCs in orange, apoptotic PCs in light rose, mature PCs in dark orange, and memory B cells in light blue. No NOX_{mRNA}^{+} B cells were observed in immature B cells, naïve B cells, plasmablasts and 'others' displayed in Figure 1.

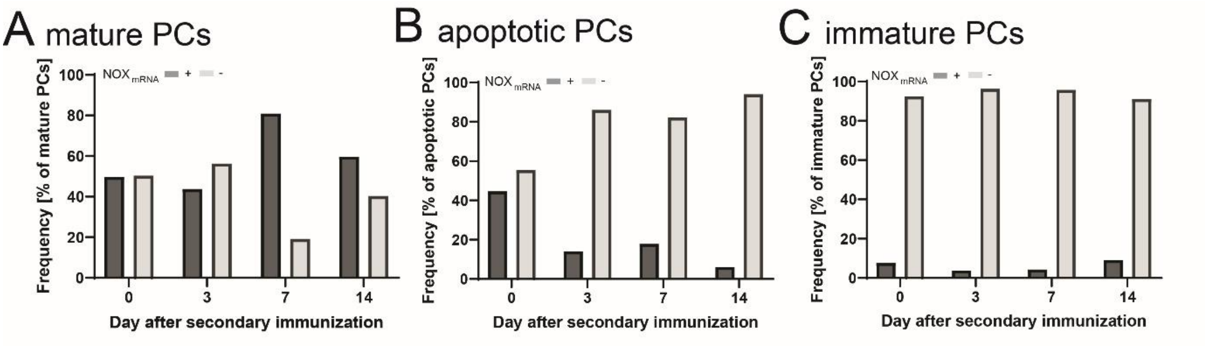

SIFigure 3: Frequencies of NOX expression in (a) mature PCs, (b) apoptotic PCs and (c) immature PCs in spleen (SP). Dark grey positive, light grey negative for NOX_{mRNA}.

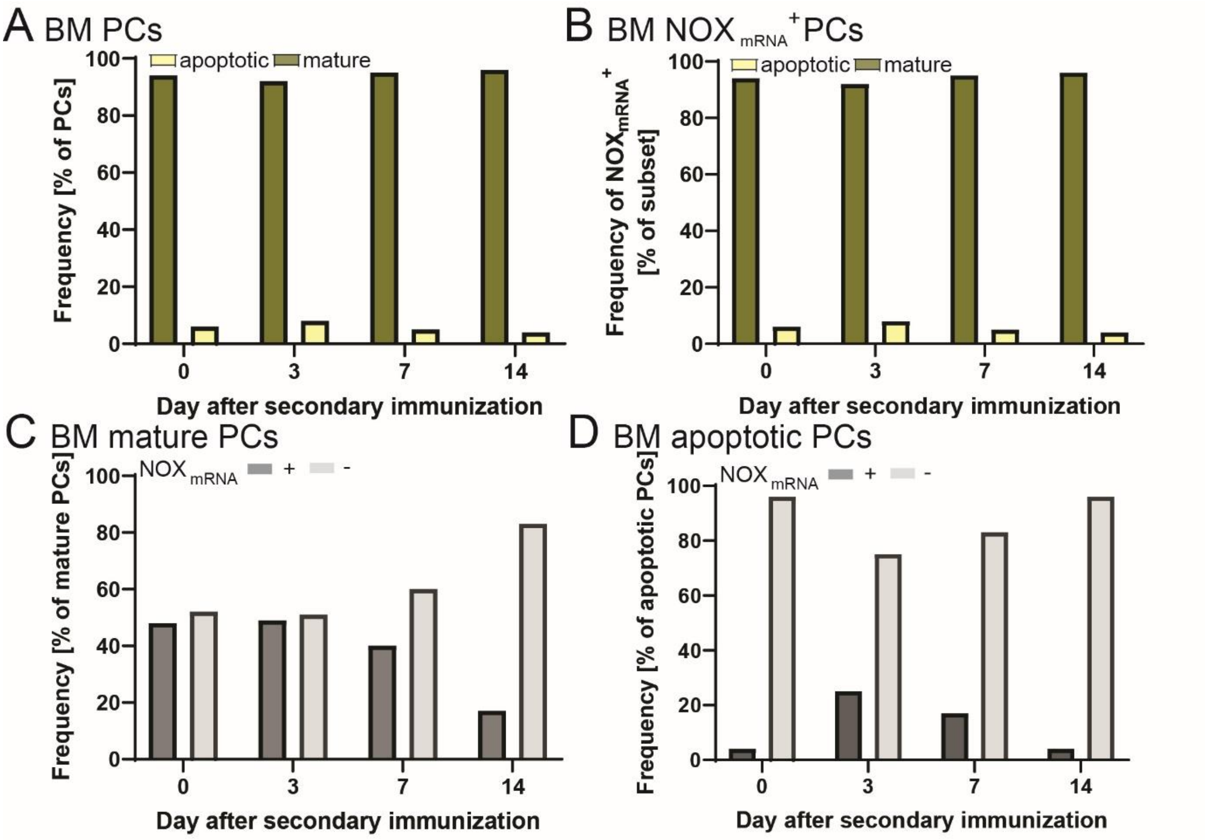

SIFigure 4: PC composition in the bone marrow (BM). (a) Frequency of apoptotic and mature PCs found in the bone marrow, relative to all PCs throughout the immune response. (b) Frequency of NOX_{mRNA}^{+} apoptotic and mature PCs related to all NOX_{mRNA}^{+} PCs. (c) Frequency of NOX_{mRNA}^{+} and NOX_{mRNA}^{-} mature PCs in BM, related to all in this category. (d) Frequency NOX_{mRNA}^{+} and NOX_{mRNA}^{-} apoptotic PCs, related to all in this category. Panels A and B depict the frequencies of cells displaying either mature (olive) and apoptotic (yellow) PC signatures, and panels C and D the frequency of NOX_{mRNA}^{+} (dark grey) and NOX_{mRNA}^{-} (light grey) in the respective subpopulation. No immature PCs and plasmablasts were found in the BM.

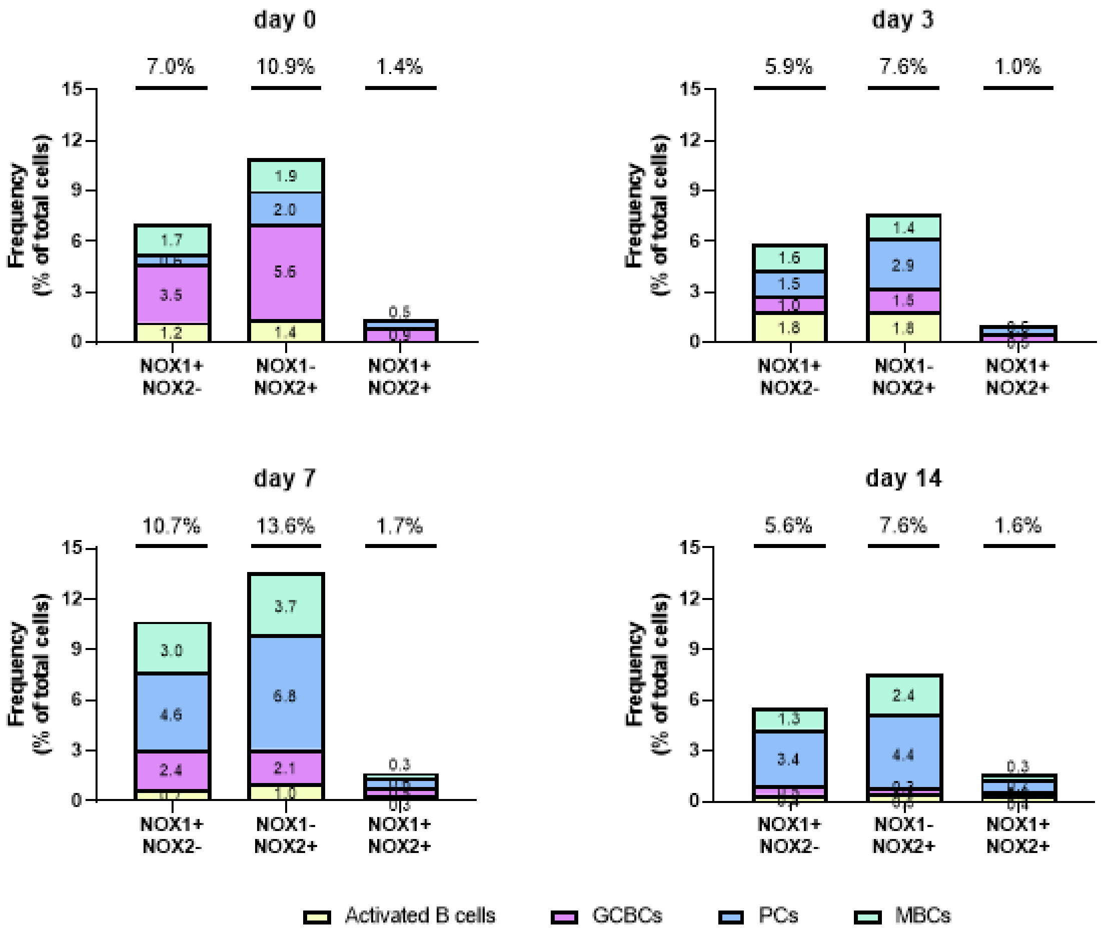

SIFigure 5: NOX isoform distributions in the NOX_{mRNA}^{+} populations over the course of the immune response and the division into B cell subpopulations. The number on top indicates the total frequency of cells expressing the respective isoforms on the distinct measurement days.

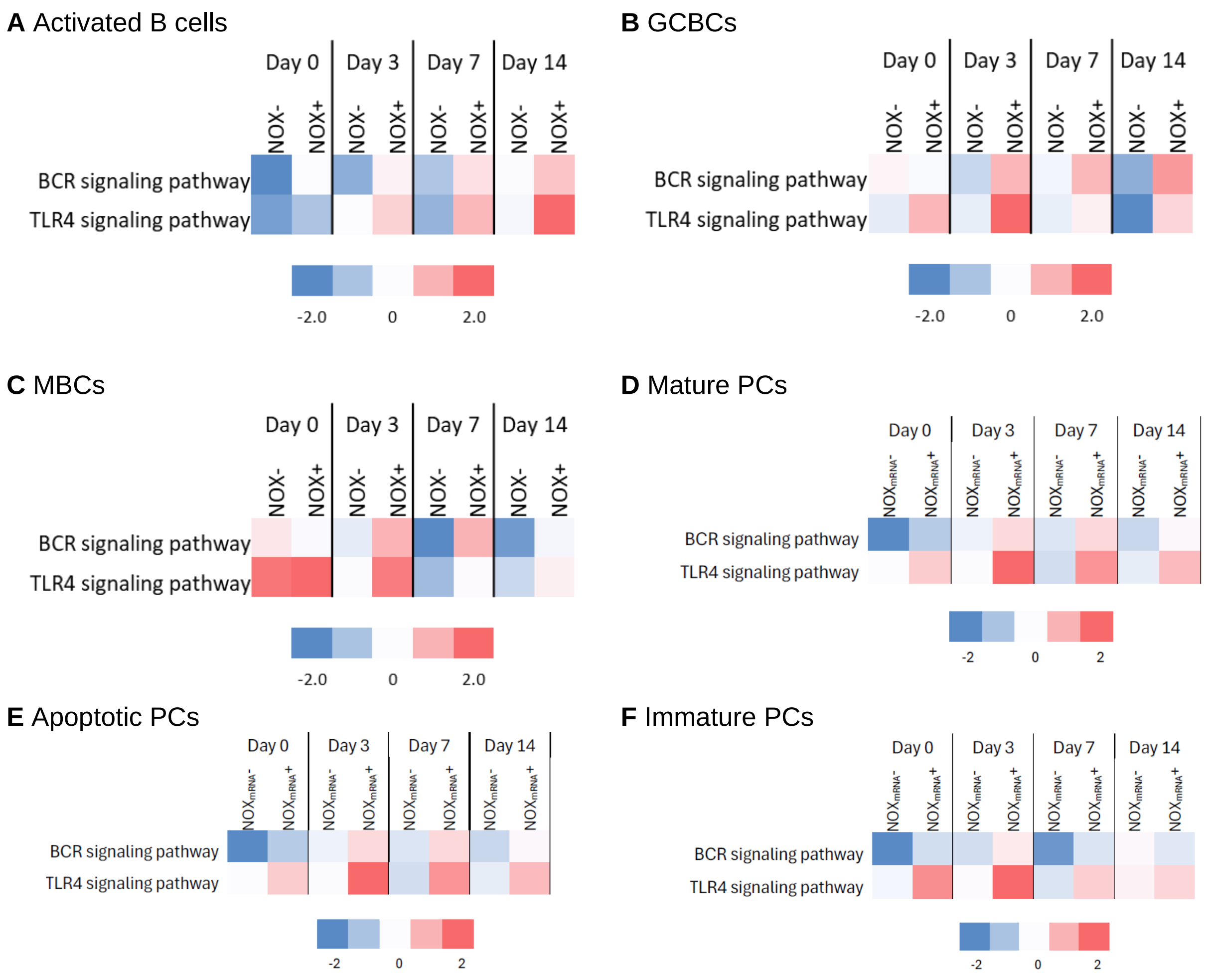

SIFigure 6: Heatmap representing the positive (red) or negative modulation (blue) of the BCR and TLR4 signaling pathways based on the average of the expression levels of their respective genes (p-value <0.05) in (a) activated B cells, (b) GCBCs, (c) MBCs, and for PC subpopulations (d) mature PCs, (e) apoptotic PCs and (f) immature PCs. In activated B cells, both signaling pathways were positively modulated in the NOX_{mRNA}^{+} subpopulation, reaching the highest activity on day 14, contrary to NOX_{mRNA}^{-} activated B cells. This trend can also be observed in the representation of both pathways in NOX_{mRNA}^{+} activated B cells in SIFigures 7-10. The BCR signaling pathway positive modulation increased over time in the NOX_{mRNA}^{+} GCBCs, whereas TLR4 signaling pathway was most positively modulated on day 3, unlike in the NOX_{mRNA}^{-} GCBCs. In NOX_{mRNA}^{+} MBCs, the BCR signaling was positively modulated on days 3 and 7, whereas it was negatively modulated in NOX_{mRNA}^{-} MBCs after antigen re-exposure. The TLR4 signaling pathway was strongly modulated in both MBC subpopulations on day 0. After re-exposure, its modulation decreased in NOX_{mRNA}^{-} MBCs, while in NOX_{mRNA}^{+} MBCs, it remained highly modulated on day 3 and decreased afterward. All PC subpopulations showed a similar behavior, namely increased TLR4 signaling, strongest early, in the NOX_{mRNA}^{+} PC subsets. BCR signaling was also higher in NOX_{mRNA}^{+} PCs. No NOX_{mRNA}^{+} were detected, therefore this comparison was not made for plasmablasts.

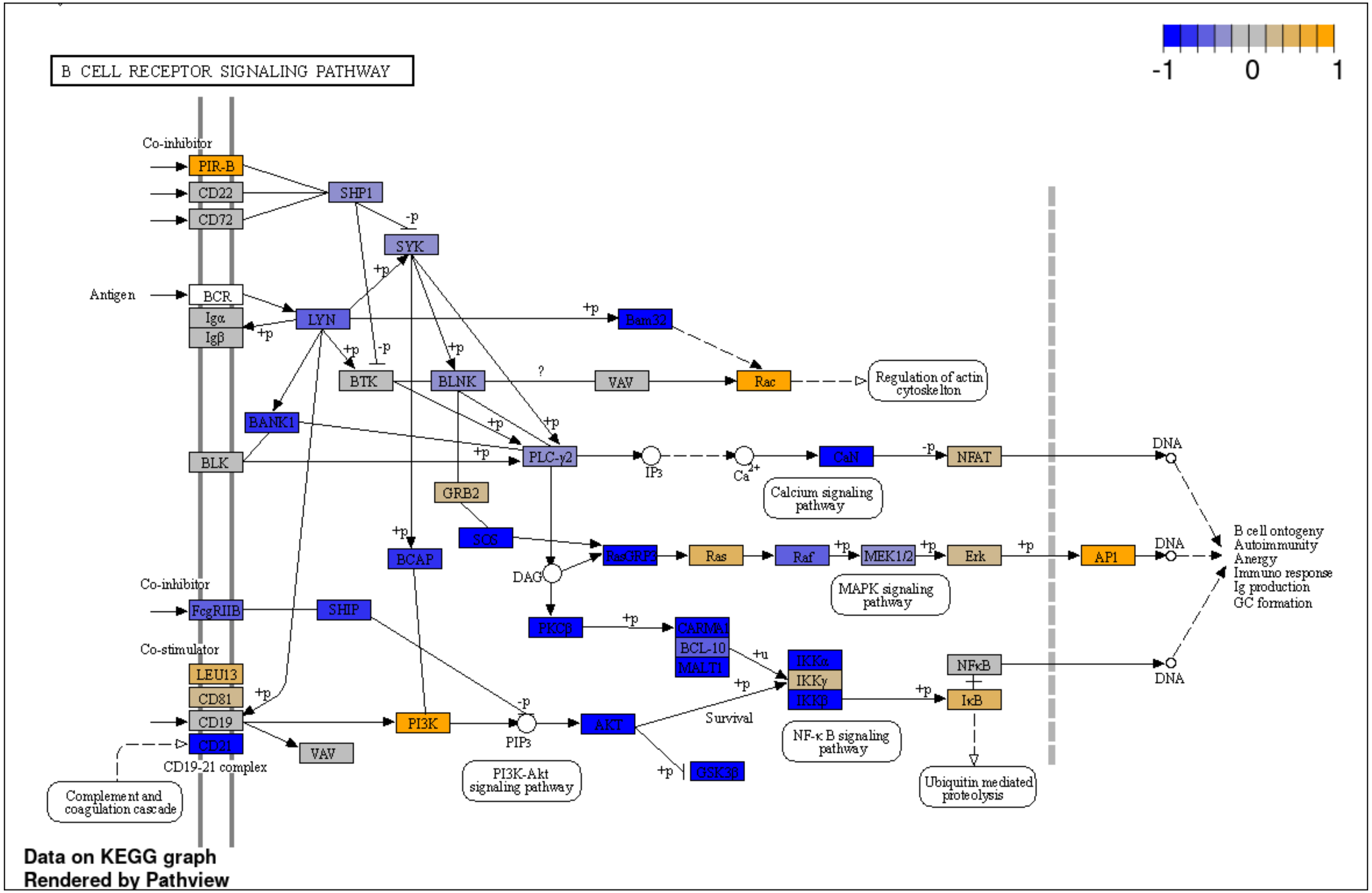

SIFigure 7: Illustration of the up-/downregulation of genes associated with the BCR signaling pathways in NOX$_{mRNA}^{+}$ activated B cells on day 0. Genes in blue: downregulated; in orange: upregulated. The chart was made with the software iDEP 0.96.

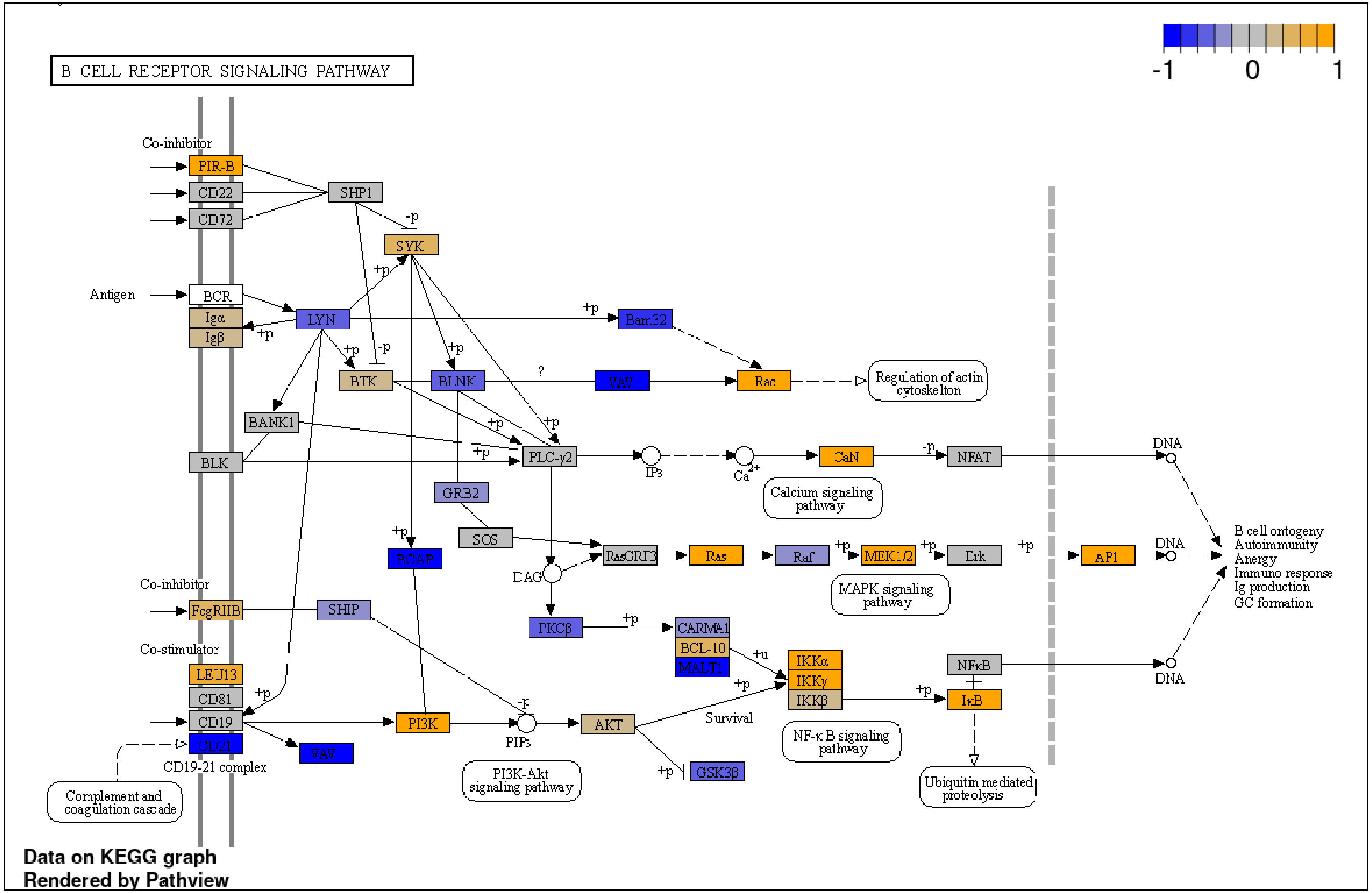

SIFigure 8: Illustration of the up-/downregulation of genes associated with the BCR signaling pathways in NOX_{mRNA}^{+} activated B cells on day 7. Genes in blue: downregulated; in orange: upregulated. The chart was made with the software iDEP 0.96.

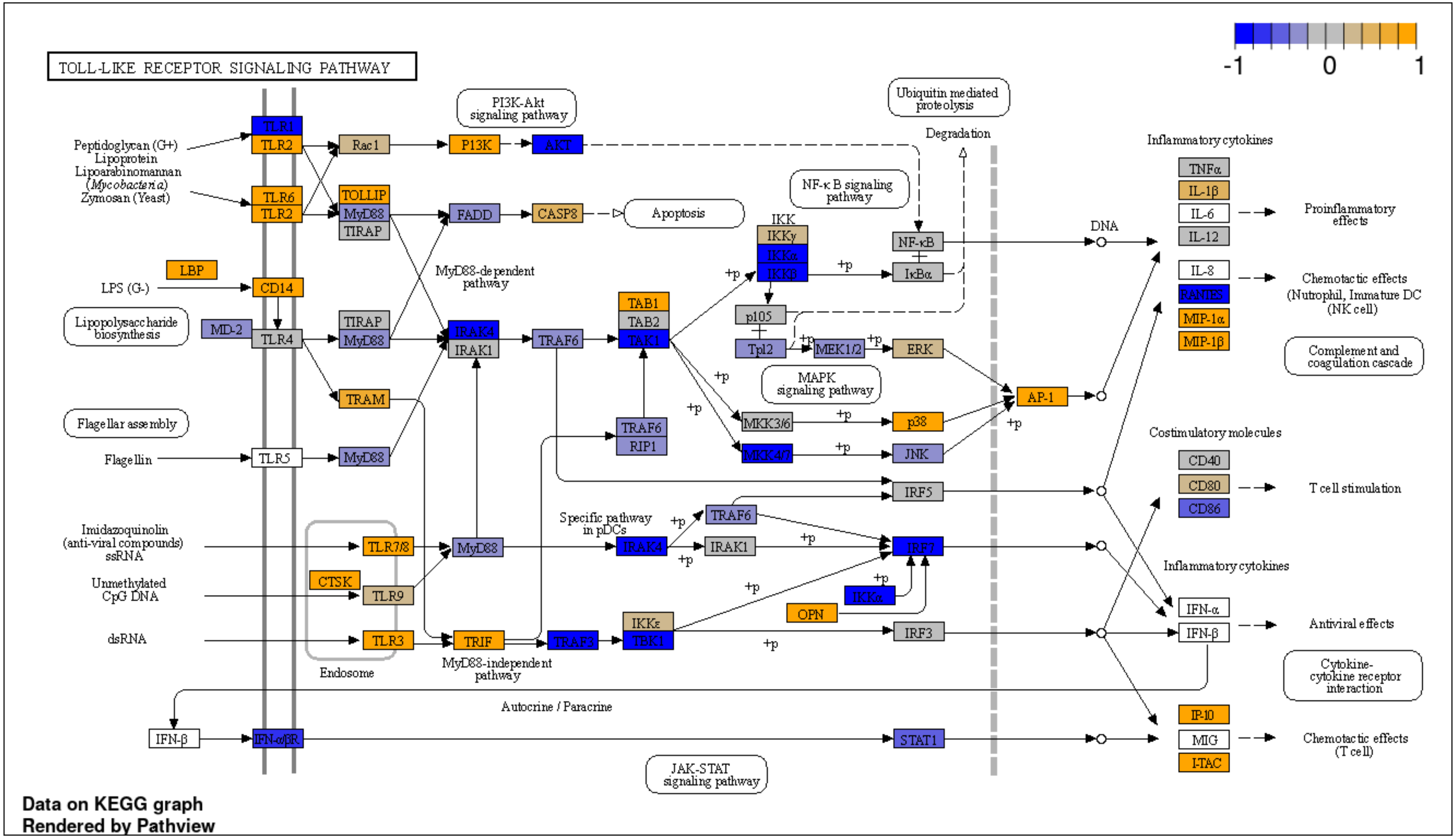

SIFigure 9: Illustration of the up-/downregulation of genes associated with the TLR4 signaling pathways in NOX$_{mRNA}^{+}$ activated B cells on day 0. Genes in blue: downregulated; in orange: upregulated. The chart was made with the software iDEP 0.96.

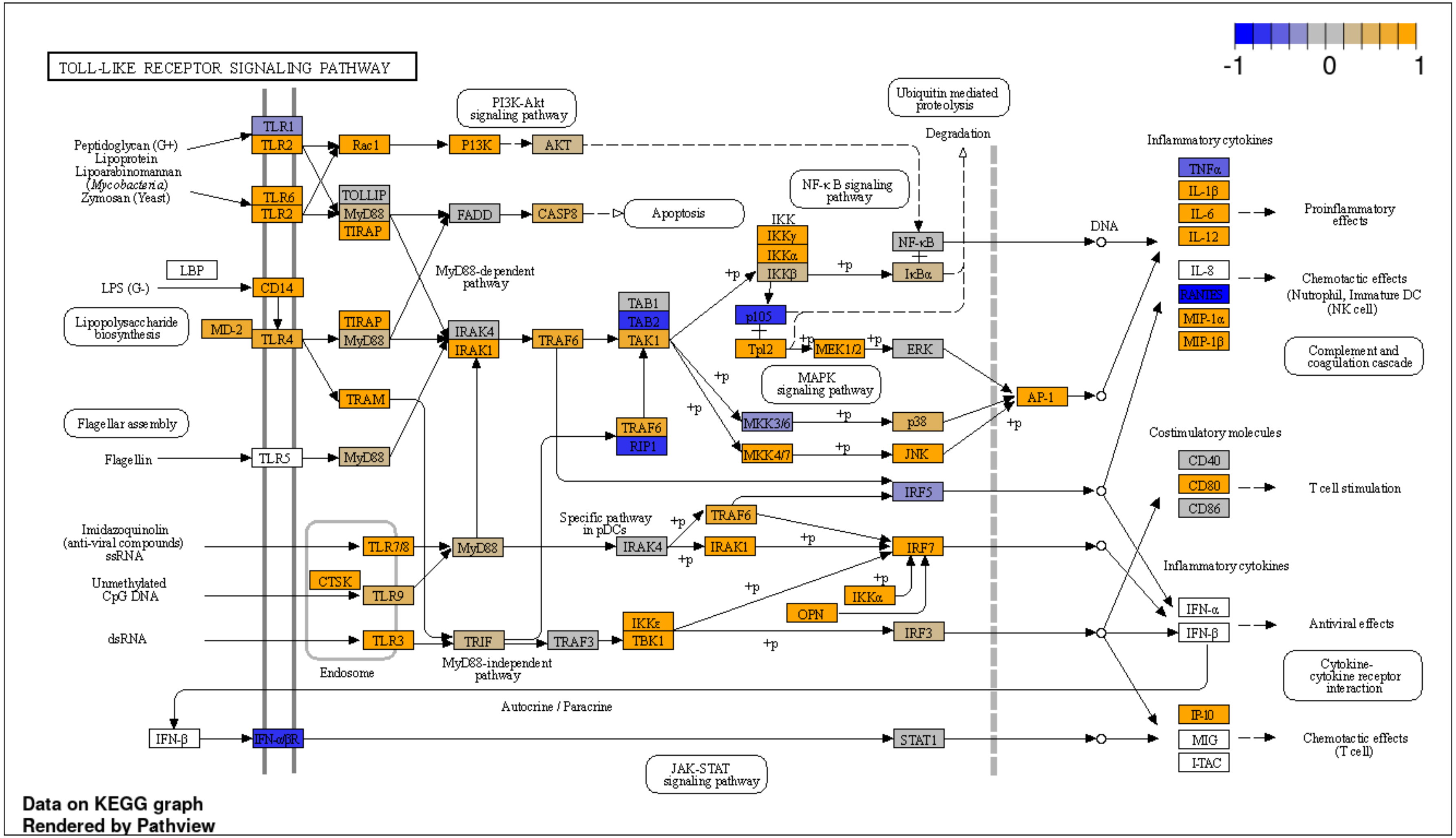

SIFigure 10: Illustration of the up-/downregulation of genes associated with the TLR4 signaling pathways in NOX$_{mRNA}^{+}$ activated B cells on day 7. Genes in blue: downregulated; in orange: upregulated. Graph was made with the software iDEP 0.96.

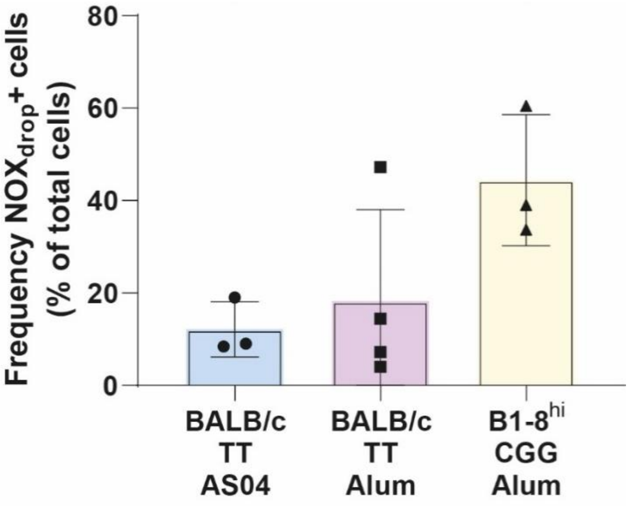

SIFigure 11: Frequency of NOX_{drop}^{+} cells obtained by immunizing BALB/c mice with TT/AS04 (blue) or TT/alum (purple) on day 7 after secondary immunization and by immunizing B1-8^{hi}YellowCaB mice with CGG/alum (yellow) on days 5-7 after a third immunization. The mean, SD and individual measurements are shown (n= 3-4).

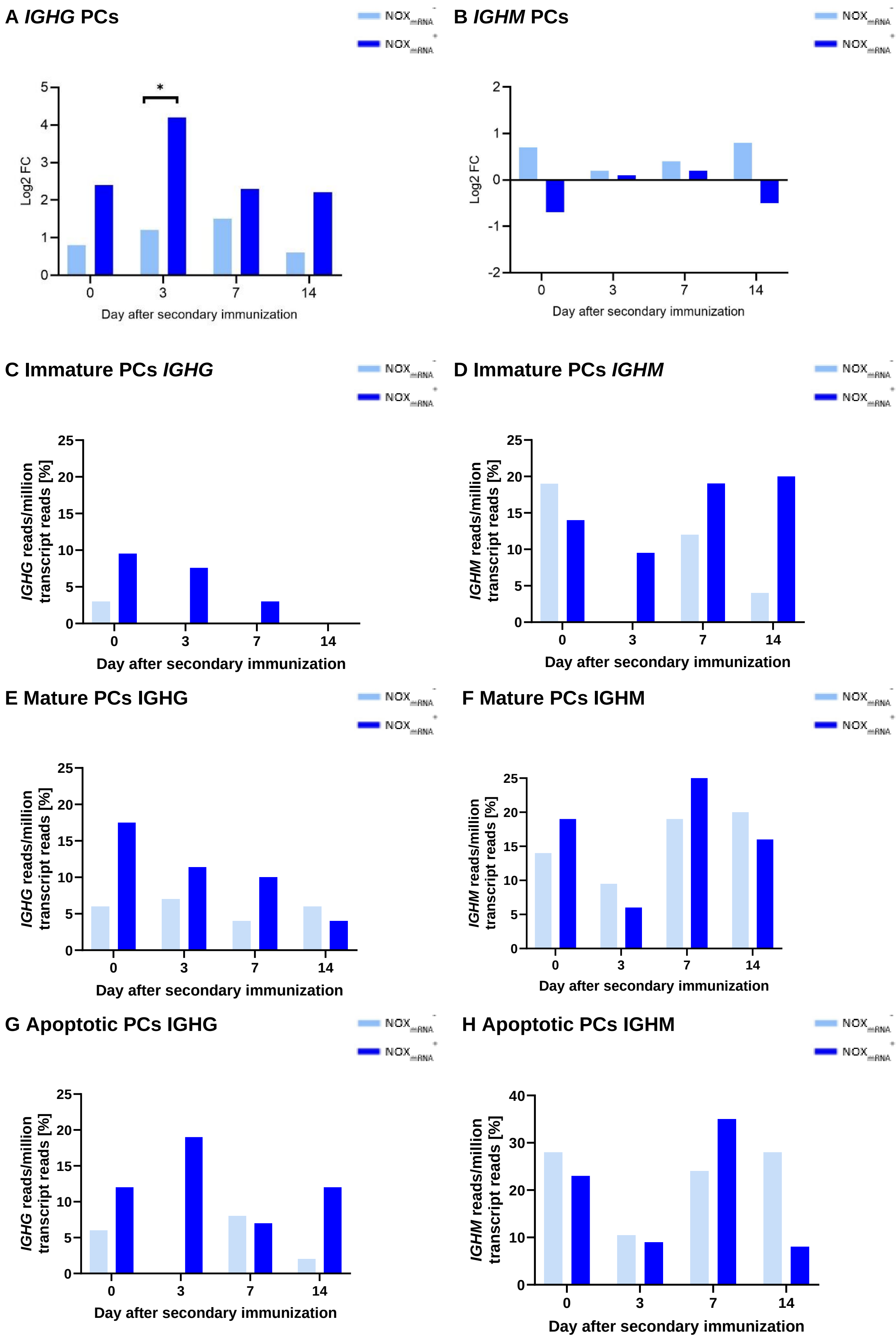
A IGHG PCs
B IGHM PCs
C Immature PCs IGHG
D Immature PCs IGHM
E Mature PCs IGHG
F Mature PCs IGHM
G Apoptotic PCs IGHG
H Apoptotic PCs IGHM
NOX_{mRNA}^{-}
NOX_{mRNA}^{+}
Log2 FC
IGHG reads/million transcript reads [%]
IGHM reads/million transcript reads [%]
Day after secondary immunization
*

SIFigure 12: Average expression levels of (a) *IGHG* and (b) *IGHM* in NOX_{mRNA}^{-} (light blue) and NOX_{mRNA}^{+} (dark blue) PCs throughout the immune response. The *IGHG* expression level differed significantly between the subpopulations on day 3 (p-value 0.03). The differences in *IGHM* expression levels were not significant during the observed period. Data presented as *IGHG* reads per total reads in percentage for the different PC subsets and their NOX status found in the data, namely immature PCs (c, d), mature PCs (e, f) and Apoptotic PCs (g,h). The data in panels C-H is shown as the percentage of IGH reads within a million transcript reads.

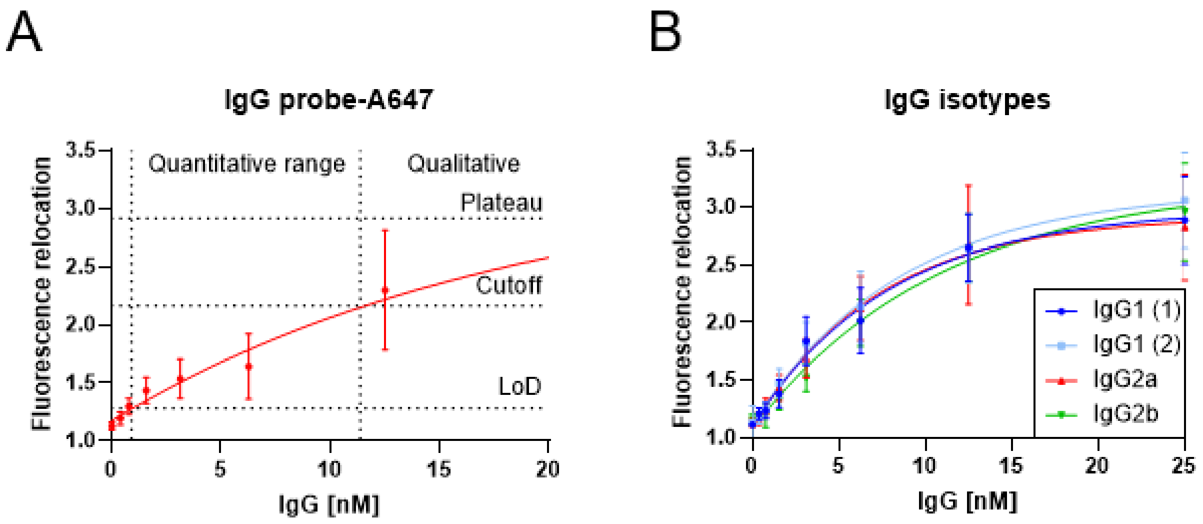

SIFigure 13: A) Calibration of the fluorescence relocation of the goat anti-mouse IgG antibody labeled with Alexa Fluor 647 as a function of the IgG concentration. The resulting quantitative range was from 9-285 IgG/s, and the limit of detection (LoD), the cutoff and the plateau are displayed in the figure. B) Fluorescence relocation of the anti-IgG antibody as a function of the IgG concentration of different isotypes, demonstrating the probe's isotype-independent binding to IgG.

SITable 2: Gene markers for ER stress and mitochondrial stress.

**ER stress**	*EIF2AK3, HSPA5, DDIT3, PPP1R15A, ATF6, ATF4*
**Mitochondrial stress**	*VDAC1, TXNIP, PPARGC1A, NFE2L2, FOXO3*
